# Estimating the cost-effectiveness of maternal vaccination and monoclonal antibodies for respiratory syncytial virus in Kenya and South Africa

**DOI:** 10.1186/s12916-023-02806-w

**Published:** 2023-03-31

**Authors:** Mihaly Koltai, Jocelyn Moyes, Bryan Nyawanda, Joyce Nyiro, Patrick K. Munywoki, Stefano Tempia, Xiao Li, Marina Antillon, Joke Bilcke, Stefan Flasche, Philippe Beutels, D. James Nokes, Cheryl Cohen, Mark Jit

**Affiliations:** 1 Department for Infectious Disease Epidemiology, London School of Hygiene & Tropical Medicine, London, UK; 2 Centre for Mathematical Modelling of Infectious Diseases, London School of Hygiene & Tropical Medicine, London, UK; 3 Centre for Respiratory Disease and Meningitis, National Institute for Communicable Diseases, Johannesburg, South Africa; 4 School of Public Health, Faculty of Health Sciences, University of the Witwatersrand, Johannesburg, South Africa; 5 Kenya Medical Research Institute (KEMRI) - Center for Global Health Research, Kisumu, Kenya; 6 KEMRI-Wellcome Trust Research Programme, KEMRI Centre for Geographical Medicine Research-Coast, Kilifi, Kenya; 7 Division of Global Health Protection, Centers for Disease Control and Prevention, Nairobi, Kenya; 8 Centre for Health Economic Research and Modelling Infectious Diseases (CHERMID), Vaccine & Infectious Disease Institute, University of Antwerp, Antwerp, Belgium; 9 School of Life Sciences, University of Warwick, Coventry, UK

**Keywords:** Respiratory syncytial virus, Cost-effectiveness analysis, Maternal vaccination, Monoclonal antibodies, Disease burden, Hospital data, ARI, SARI

## Abstract

**Background:**

Respiratory syncytial virus (RSV) causes a substantial burden of acute lower respiratory infection in children under 5 years, particularly in low- and middle-income countries (LMICs). Maternal vaccine (MV) and next-generation monoclonal antibody (mAb) candidates have been shown to reduce RSV disease in infants in phase 3 clinical trials. The cost-effectiveness of these biologics has been estimated using disease burden data from global meta-analyses, but these are sensitive to the detailed age breakdown of paediatric RSV disease, for which there have previously been limited data.

**Methods:**

We use original hospital-based incidence data from South Africa (ZAF) and Kenya (KEN) collected between 2010 and 2018 of RSV-associated acute respiratory infection (ARI), influenza-like illness (ILI), and severe acute respiratory infection (SARI) as well as deaths with monthly age-stratification, supplemented with data on healthcare-seeking behaviour and costs to the healthcare system and households. We estimated the incremental cost per DALY averted (incremental cost-effectiveness ratio or ICER) of public health interventions by MV or mAb for a plausible range of prices (5–50 USD for MV, 10–125 USD for mAb), using an adjusted version of a previously published health economic model of RSV immunisation.

**Results:**

Our data show higher disease incidence for infants younger than 6 months of age in the case of Kenya and South Africa than suggested by earlier projections from community incidence-based meta-analyses of LMIC data. Since MV and mAb provide protection for these youngest age groups, this leads to a substantially larger reduction of disease burden and, therefore, more favourable cost-effectiveness of both interventions in both countries. Using the latest efficacy data and inferred coverage levels based on antenatal care (ANC-3) coverage (KEN: 61.7%, ZAF: 75.2%), our median estimate of the reduction in RSV-associated deaths in children under 5 years in Kenya is 10.5% (95% CI: 7.9, 13.3) for MV and 13.5% (10.7, 16.4) for mAb, while in South Africa, it is 27.4% (21.6, 32.3) and 37.9% (32.3, 43.0), respectively.

Starting from a dose price of 5 USD, in Kenya, net cost (for the healthcare system) per (undiscounted) DALY averted for MV is 179 (126, 267) USD, rising to 1512 (1166, 2070) USD at 30 USD per dose; for mAb, it is 684 (543, 895) USD at 20 USD per dose and 1496 (1203, 1934) USD at 40 USD per dose. In South Africa, a MV at 5 USD per dose would be net cost-saving for the healthcare system and net cost per DALY averted is still below the ZAF’s GDP per capita at 40 USD dose price (median: 2350, 95% CI: 1720, 3346). For mAb in ZAF, net cost per DALY averted is 247 (46, 510) USD at 20 USD per dose, rising to 2028 (1565, 2638) USD at 50 USD per dose and to 6481 (5364, 7959) USD at 125 USD per dose.

**Conclusions:**

Incorporation of new data indicating the disease burden is highly concentrated in the first 6 months of life in two African settings suggests that interventions against RSV disease may be more cost-effective than previously estimated.

**Supplementary Information:**

The online version contains supplementary material available at 10.1186/s12916-023-02806-w.

## Background

Respiratory syncytial virus (RSV) is a globally widespread [1, 2] endemic virus, which is the most common pathogen in children diagnosed with acute lower respiratory infections (ALRI) [3–5]. Most symptomatic and severe cases are in children younger than 5 years, with infants being the most affected, as most severe cases occur in the first year of life. The disease burden is particularly high in low- and middle-income countries (LMICs), where the majority of global RSV-attributable hospitalisations and deaths are concentrated [3], with latest estimates suggesting over 62% of RSV-associated hospitalisations and nearly all deaths globally in LICs and LMICs [3].

Both maternal vaccination (MV) and monoclonal antibodies (mAb) may confer protection against RSV disease in early life. There have been multiple efforts to develop a maternal vaccine [6]. In 2020, the maternal vaccine candidate (ResVax) showed protection against RSV LRTI-associated hospitalisation and severe hypoxemia [7] but did not meet its primary clinical endpoint across all study sites due to low (slightly below 50%) efficacy in the arm of the trial conducted in the USA. However, more recently another MV (PF-06928316 or RSVpreF) showed higher efficacy figures in phase 3 clinical trials, both against RSV-associated medically attended lower respiratory tract infection (MA-LRTI, 57.1%, 95% CI: 14.7 to 79.8) and against severe MA-LRTI (81.8%, 95% CI: 40.6 to 96.3).

Similarly, recent pooled phase 3 clinical trial data for the mAb candidate nirsevimab (Beyfortus) showed above 75% efficacy both against RSV MA-LRTI 79.5 (65.9 to 87.7) and RSV LRTI hospitalisation 77.3% (50.3 to 89.7) [8–10].

With these preventive biologics against RSV infection showing promise and possibly becoming commercially available in the years to come, it is important to assess the cost-effectiveness of public health interventions via vaccines or monoclonal antibodies for infants. This is especially important in the context of LMICs which have the highest burden but also face financial constraints and competing demands on healthcare resources from other diseases. The only currently available prophylactic RSV treatment (palivizumab, a monoclonal antibody administered by monthly injections over the RSV season) is in use only in a few high-income countries where it is recommended exclusively to high-risk groups due to its high cost [11]. Because maternal and next generation (long half-life) monoclonal antibodies have a duration of protection of 3–6 months, the cost-effectiveness of both vaccines and monoclonal antibodies depends on the age distribution of RSV burden in infants. The monthly resolution of the incidence data of the current study and the separate estimates for all and severe RSV disease allow for a more detailed analysis than the larger age brackets used for infants in previous analyses [3].

In this study, we provide updated estimates for the cost-effectiveness of MV or mAb-based public health interventions to prevent RSV disease in children under 5 years of age in two African countries, one lower middle income (Kenya) and the other upper middle income (South Africa). A previous study by Li et al. [12] used estimates for RSV incidence in the community and in hospital from Shi et al. [3] to quantify the cost-effectiveness of these interventions in 72 Gavi-eligible countries, while accounting for uncertainty in parameters by using probabilistic sensitivity analysis. We are able to refine this analysis by using recently available finely (monthly) age-stratified estimates for RSV-associated respiratory infections, hospitalisations, and deaths in Kenya and South Africa. We also use new data from the same sources on country-specific cost estimates for both countries.

## Methods

### Study population and data collection

The age-stratified incidence of RSV-associated non-severe (ARI) and severe (SARI) acute respiratory illness was estimated for both countries. The methodology for obtaining and calculating incidence is described in detail in the accompanying papers (Moyes et al. 2022 [13], Nyawanda et al. 2023 [14]) and briefly described below.

**Fig. 1:**
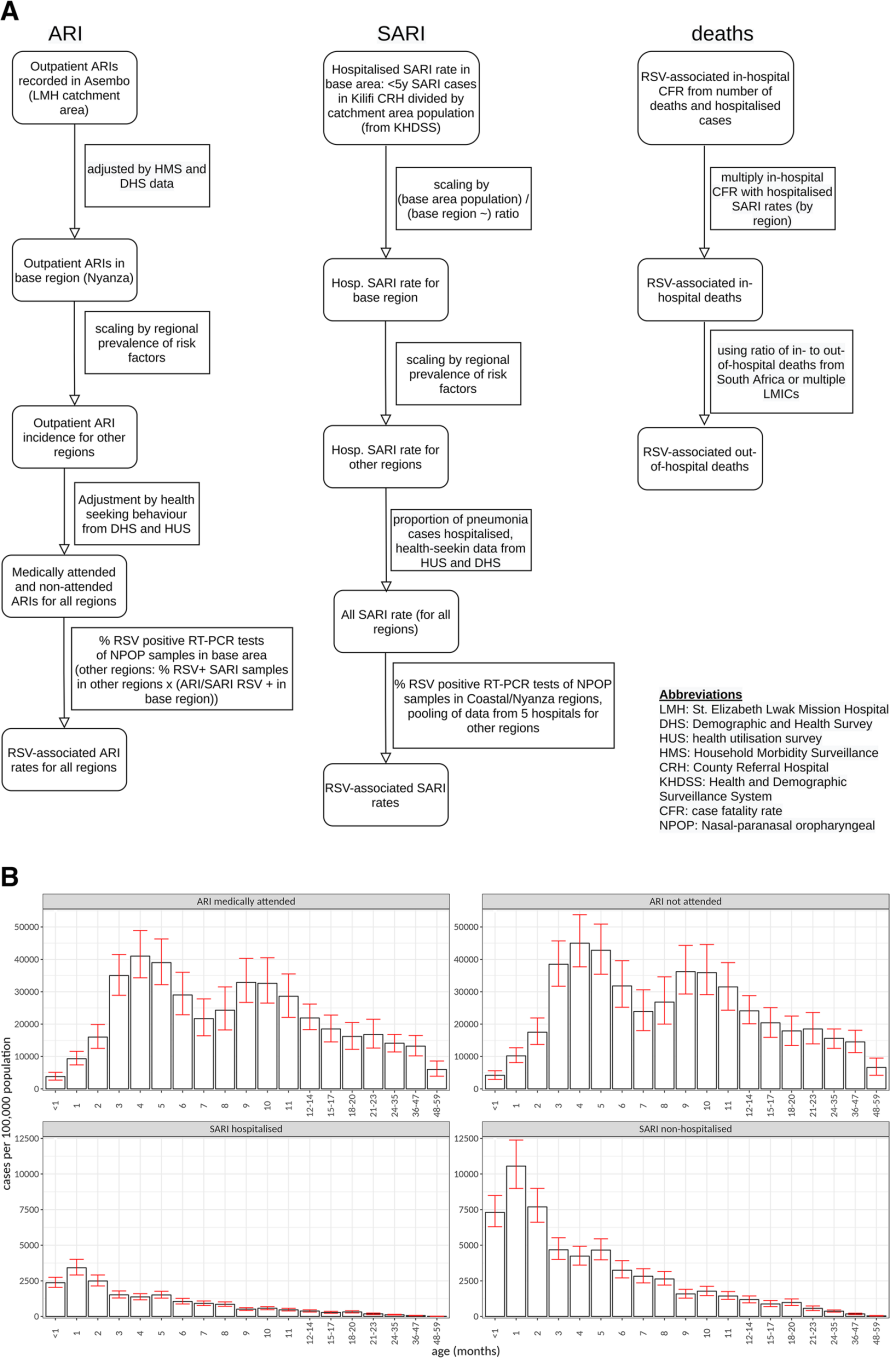
**A** Methods used for incidence rates of ARI, SARI and deaths in Kenya. **B** Estimated RSV-associated ARI and SARI rates per 100,000 person-years in Kenya by age group and medical status. Error bars show 95% CI values

**Fig. 2:**
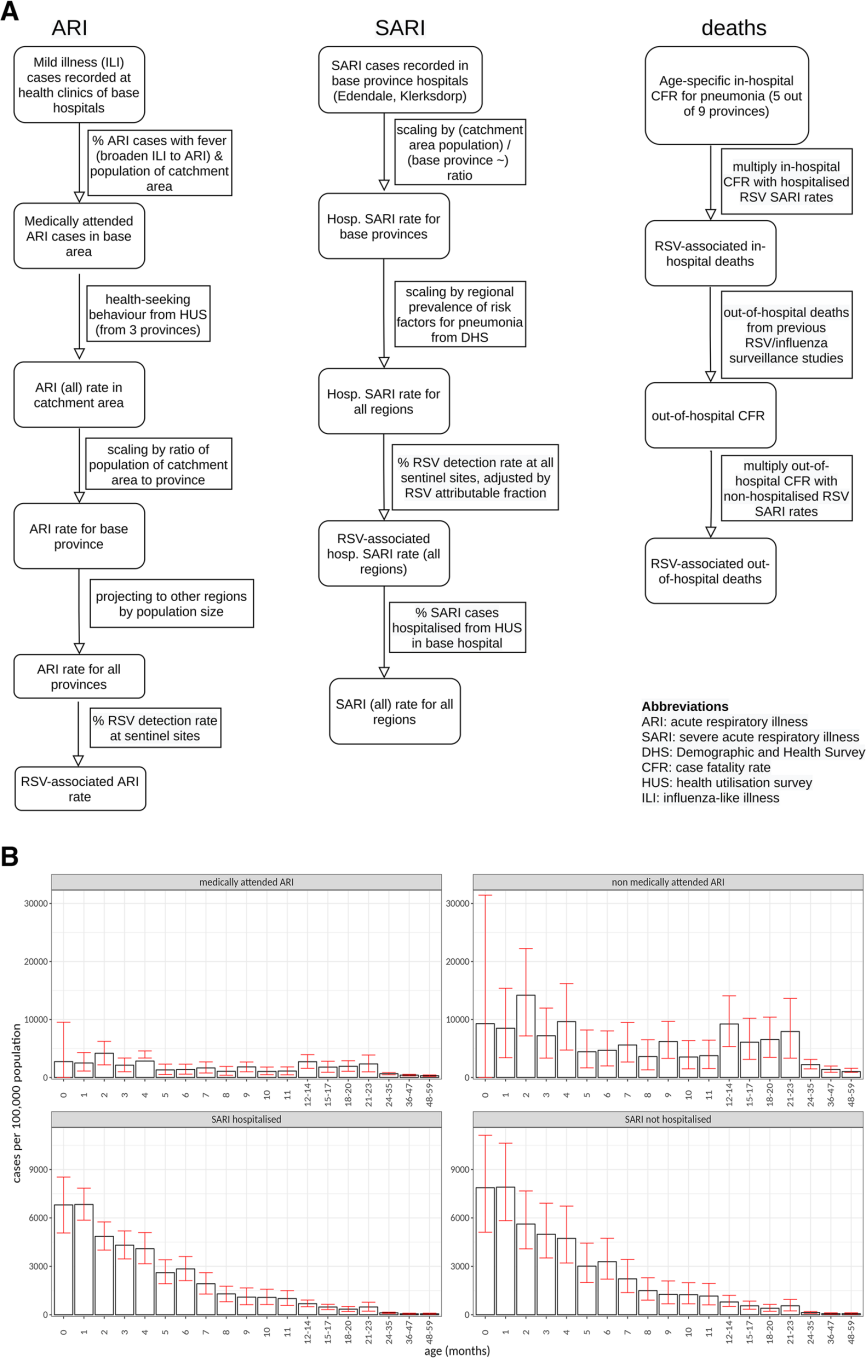
**A** Methods used for incidence rates of ARI, SARI and deaths in South Africa. **B** Estimates of RSV-associated ARI and SARI rates per 100,000 person-years in South Africa by age group and medical status. Error bars show 95% CI values. ARI rates shown are before applying the adjustment described in the “Methods” section that aligns the South African ILI rates with the ARI definition

The methods used for collecting and processing data are described in detail in the accompanying papers by Nyawanda 2023 [14] and Moyes 2023 [13]. We summarise the methods in Figs. 1A and 2A, with further details in Additional file 1: SI Table 1 and Additional file 1: SI Methods 1.2.

### Estimates of RSV-associated deaths in- and out-of-hospital

**Fig. 3:**
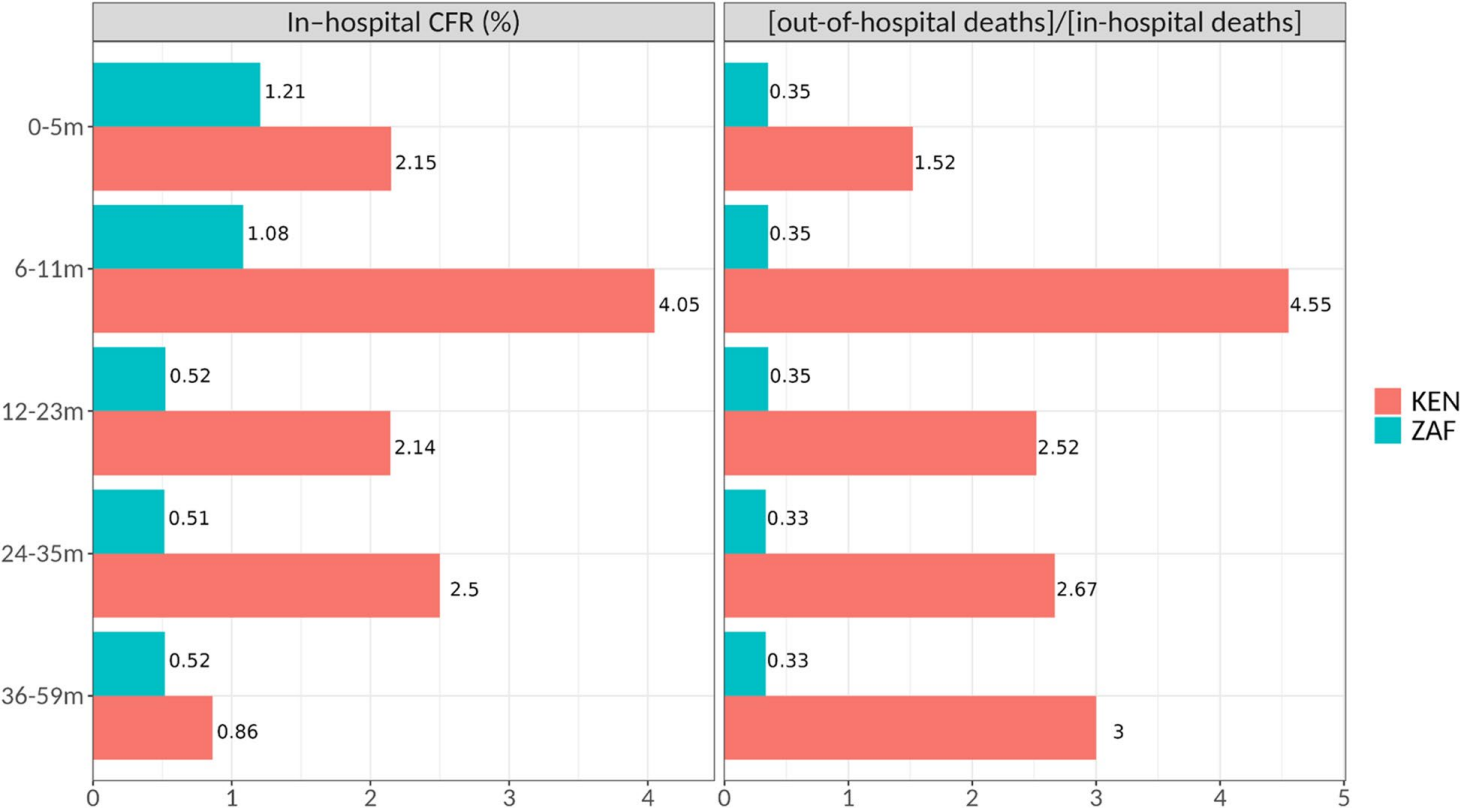
In-hospital case fatality rate (CFR) and the ratio of out-of-hospital to in-hospital deaths used to calculate RSV-associated deaths. CFR values are new data described in the accompanying paper by Nyawanda et al. [14] for Kenya and from [15] for South Africa. The out-of/in-hospital deaths ratio was obtained from [5] for Kenya and from [17] for South Africa

In-hospital deaths were estimated by applying CFR values (Fig. 3) in 6-month bands under the age of one year (and in 12–24-month bands from 1 to 5 years of age) to the hospitalisation (SARI) data. In the case of Kenya, the CFR values are new estimates of the accompanying paper Nyawanda et al. [14]; in the case of South Africa, they are from [15] (see accompanying paper [13]). Out-of-hospital deaths were then estimated by applying their previously estimated ratio to in-hospital deaths (figures from [5] for KEN, [16] for ZAF). The more than fourfold estimated under-reporting in the 6–11-month period in Kenya leads to a second peak of deaths at the age of 6–8 months, which the interventions do not cover anymore when assuming they have a uniform efficacy for 3 (MV) and 5 (mAb) months, respectively, and no effect afterwards.

We performed a sensitivity analysis where we lumped our estimates of deaths under 1-year of age (where it had monthly resolution) in 3 or 6 month age brackets, to test if propagating the monthly resolution of hospitalisations substantially affects our results.

We also performed a sensitivity analysis where efficacy exponentially decays (Additional file 1: SI Figure 2, Additional file 1: SI Table 3, Additional file 1: SI Methods 2.2) instead of having a binary effect, to explore how this changes cost-effectiveness outcomes.

### Calculation of disease burden, cost, and cost-effectiveness

In this study, we adopted the McMarcel analytical framework from [12], modifying it to incorporate observed age-specific incidence rates of ARIs, SARIs, and deaths, as well as treatment costs of RSV disease from Kenya and South Africa, distinguishing disease episodes by whether they involved medical treatment.

The cost-effectiveness of passive immunisation (via MV or mAb) to prevent RSV disease after birth in 72 Gavi-eligible countries was estimated by Li et al. [12] using the static model McMarcel (Multi-Country Model Application for RSV Cost-Effectiveness poLicy), made available as an R package [18]. This model used country-specific estimates of total RSV incidence, hospitalisation probabilities, and hospital and community case fatality ratios (CFR) based on the 2017 Shi et al. meta-analysis [3] of global RSV burden. These estimates were used in a probabilistic sensitivity analysis where incidence and CFR values are generated from distributions around the age-specific mean values. The disease burden and the associated costs were then reduced proportionally to the assumed efficacy of MV and mAb, while also calculating the cost of the intervention. Since all measures are probabilistically treated, the model produces a distribution for the cost effectiveness of the interventions as well.

For South Africa, we estimated the cost of outpatient care (25 USD, 95% CI: 18.3–31.8) and the cost of hospitalisation by age group (Additional file 1: SI Figure 1) (Moyes et al. 2022 [13]). Hospitalisation costs include facility fees and the cost of consultations, comprising approximately 60–80% of the total cost, while in age groups where ICU visits were observed, they comprise approximately 20% of the total in-patient cost. Approximately 4 children under 5 years of age were enrolled during RSV seasons (2 outside the season) per week at each site for cost estimation. Frequency of ICU visits and lengths of stay were averaged in 3-month age bands. In South Africa hospital care for infants is free of charge, therefore, costs of RSV disease requiring hospitalisation are largely absorbed by the healthcare system.

In the case of Kenya, only the total cost to the healthcare system at the Siaya site (Siaya County Referral Hospital) was available. We used costs from Siaya because at the other available site (Kilifi), costs are largely subsidised from international sources and therefore do not reflect typical costs to the healthcare system. We used the ratio of inpatient to outpatient costs (per episode) of RSV-associated illness from Malawi [19], which was 4.9, and the number of inpatient and outpatient episodes to estimate inpatient and outpatient costs separately from the total cost.

In Kenya, a large proportion of the total cost of hospitalisation falls on households. These costs comprise both direct out-of-pocket payments for drugs and transportation and loss of income due to absence from work. In the cost-effectiveness analysis, we summed the costs to the healthcare system and those to households (Additional file 1: SI Table 2) to calculate the cost of disease burden and its reduction by interventions.

### Fitting efficacy estimates and using different models of efficacy

For the base case, we updated previous (assumed) efficacy estimates [12] with the most recent results of phase 3 clinical trials of a maternal vaccine candidate [20] and the monoclonal antibody nirsevimab (Beyfortus) [8–10, 21] (Table 1). We fitted the mean values and 95% credible intervals of efficacy against RSV-associated medically attended LRTI, hospitalisation, and severe disease with beta distributions to reflect the uncertainty in efficacy estimates (Additional file 1: SI Table 4, Additional file 1: SI Methods 2.1).

**Table 1:** Efficacy figures used for cost-effectiveness analysis. For efficacy against hospitalisations by MV, the efficacy against severe MA-LRTI was used. For efficacy against deaths by mAb, the efficacy against RSV LRTI hospitalisations was used

Product type	End point	Mean efficacy (95% CI)	Source
MV (maternal vaccine)	Medically attended lower respiratory tract illness (MA-LRTI), first 90 days of life	57.1 (14.7 to 79.8)	[20]
MV	Severe MA-LRTI, first 90 days of life	81.8 (40.6 to 96.3)	[20]
mAb (monoclonal antibodies)	RSV MA-LRTI, first 151 days of life	79.5 (65.9 to 87.7) (pooled estimate from [21])	[8, 9, 21]
mAb	RSV LRTI hospitalisations, first 151 days of life	77.3 (50.3 to 89.7)	[8, 9, 21]

In the base case presented in the main text, we assumed both MV and mAb to have a fixed duration of protection (3 months for MV and 5 months for mAb) and to immediately fall to zero beyond that point. We performed a scenario sensitivity analysis with regard to this assumption by assuming that efficacy decays exponentially over time, with the same average value as observed in the clinical trials over the 0–90 day (MV) and 0–150 day (mAb) periods and half-life values identical (or as close as possible) to the reported values (Additional file 1: SI Table 2 and 3, Additional file 1: SI Methods 2.2). Exponential waning, therefore, implies that efficacy is highest immediately after birth and gradually wanes with time.

### Probabilistic sensitivity analysis

Gamma distributions were fitted to the 95% CIs of the incidence of RSV-associated ARI, SARI, and death from both countries to reflect the uncertainty of estimates. Our dataset distinguishes between non-severe (ARI for Kenya and ILI for South Africa, Additional file 1: SI Table 1) and severe (SARI) cases and also between cases that were medically attended or not. In addition, we also estimate community incidence of severe cases (non-hospitalised SARIs), inferred from the hospital-based incidence via estimates of healthcare-seeking behaviour (Figs. 1A and 2A).

We performed a probabilistic sensitivity analysis, whereby 5000 samples were generated per age group from the distributions of the input parameters with uncertainty. These rates were then multiplied by health utility measured in disability-adjusted life years (DALYs) for non-severe and severe disease (Additional file 1: SI Table 2) to calculate the total disease burden. Similarly, incidence rates were multiplied by unit cost estimates to calculate total medical costs.

We first calculated mean and median values with corresponding credible intervals (50%, 95%) for disease burden in terms of case numbers (non-hospitalised and hospitalised cases, deaths) and in units of DALYs, as well as the costs before the interventions.

In the next step, we calculated the effect of interventions by using the distributions generated from efficacy figures and computing the number of cases (non-hospitalised, hospitalised and deaths) averted, DALYs averted and the medical cost averted. As the measure of cost-effectiveness, we computed the incremental cost of the intervention (intervention cost minus medical costs averted) and then calculated the incremental cost per DALY averted (ICER) of each intervention. We used a 3% discounting rate both for health outcomes and costs, while costs are in units of 2020 US dollars (USD).

For coverage, we used the latest literature estimate [22] of the inferred level of coverage for MV and mAb-based interventions against RSV: 61.6% for Kenya and 75.2% for South Africa. These figures were based on the timing and coverage for several ANC visits and hence are lower than current ANC-3 coverage levels (KEN: 69.6%,: 81.57%); therefore, they can be seen to represent a conservative estimate. We note that while the reduction in disease burden scales linearly with the coverage level (Additional file 1: SI Figure 3), the cost effectiveness of the interventions does not change with different levels of coverage, as in our model coverage scales the cost and the intervention effect by the same factor.

As a metric of cost-effectiveness, we used the ratio of the incremental cost to DALYs averted (ICER, incremental cost-effectiveness ratio). To estimate the cost-effectiveness of interventions, we used a range of realistic values for the price of a complete course of MVs (5 to 50 USD) and mAbs (10 to 125 USD). The lowest prices used are close to baseline assumptions in the Li 2020 [12] cost-effectiveness study on RSV interventions in LMICs and yield ICER values less than one-tenth of the GDP/capita of the two countries (see the “Results” section). The analysed price range was capped at the level where the median ICERs exceeded the GDP/capita of the two countries.

## Results

### Disease burden impact

**Fig. 4:**
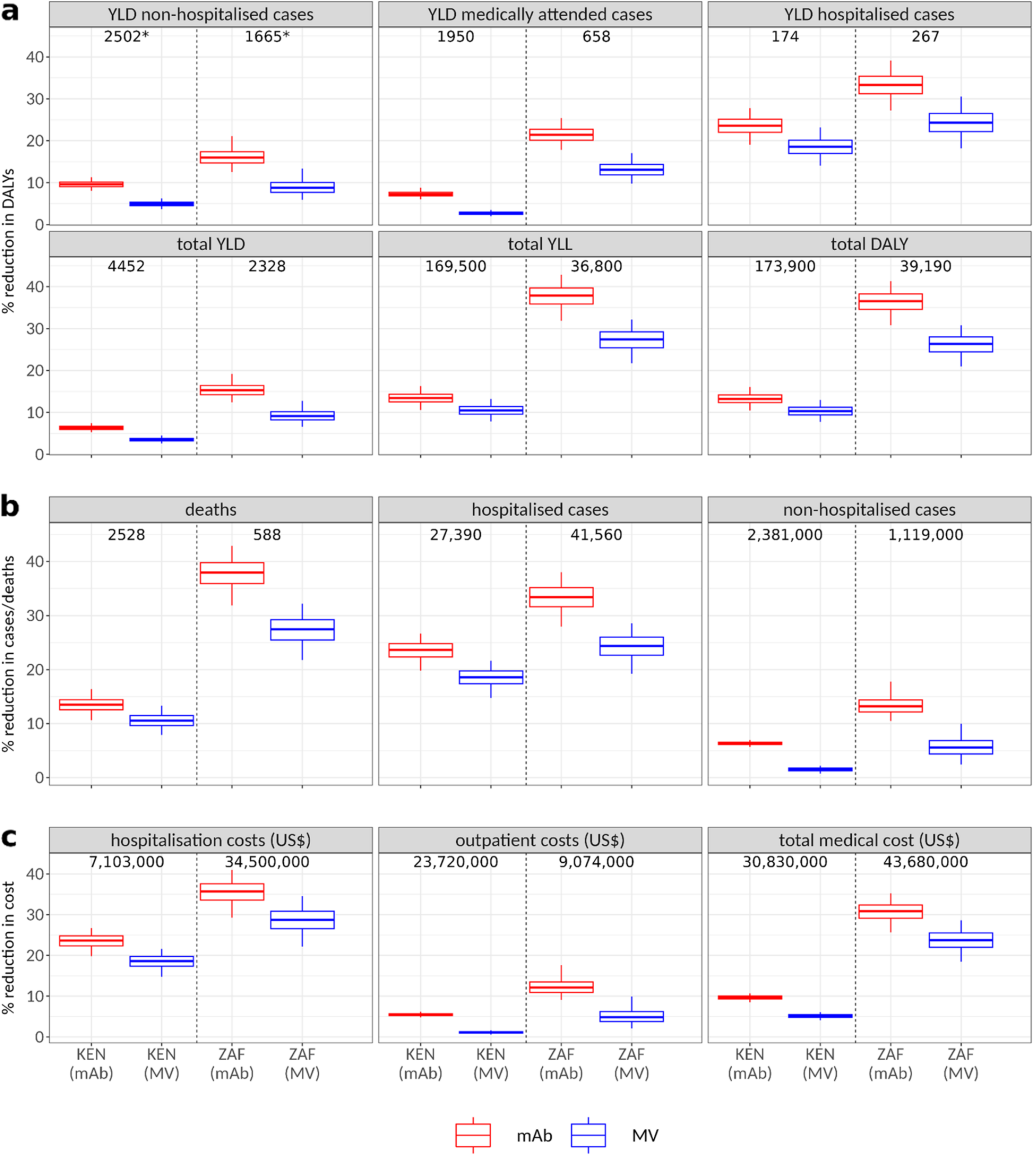
**A** Percentage reduction (*y*-axis) in non-hospitalised and hospitalised RSV cases and RSV-associated deaths in response to maternal vaccination or monoclonal antibody use. Solid horizontal lines show the reduction as a percentage of the variables’ pre-intervention median values, which are displayed by the numbers at the top of panels (the pre-intervention values are the same for MV and mAb). Horizontal lines show the median values. Boxes show 50% and whiskers the 95% credible intervals. **B** Percentage reduction in subtypes of disease burden (DALYs) in response to maternal vaccination or monoclonal antibody campaigns. **C** Percentage reduction in costs of disease burden (USD) in response to maternal vaccination or monoclonal antibody campaigns

The total burden expressed in DALYs is dominated by deaths. In both countries, approximately 95% of the total DALYs (Fig. 4) are due to life years lost because of death.

According to our hospitalisation estimates, out of all SARI episodes (under 5 years of age), in Kenya, 45% are in infants aged ≤ 3 months, and 65% are in infants ≤ 6 months. In South Africa, 48% are in infants aged ≤ 3 months and 62% in infants ≤ 6 months. In both countries, we observed peak incidence at 1 month of age for SARI episodes (Figs. 1 and 2).

In-hospital deaths were estimated by applying CFR values to hospitalisations and subsequently the ratio of out-of- to in-hospital deaths (Fig. 3). Following this procedure, we estimated that 34% of deaths in Kenya occur in the first 6 months of life, while this figure is 73% in South Africa. The age-specific ratios of out-of to in-hospital deaths used for Kenya were estimates for all LMICs in [5] with a higher ratio (4.55×) for the 6–11m age band, leading to more estimated deaths above the age of 6 months.

We note that some of our ARI/SARI incidence data also had to be inferred and are estimates rather than measured cases. Specifically, RSV testing for ARI and SARI rates were not available in Kenya in all regions, so the base region positivity rate was projected to get the national average (Fig. 1A). Non-hospitalised SARIs in Kenya were obtained by applying the hospitalisation rate of pneumonia to the hospitalised SARI rate.

As a consequence of the relative concentration of severe cases and deaths in the first few months of life where both MV or mAb have their effect, the interventions can lead to a substantial reduction in disease burden. The reduction in the disease burden will linearly scale with the coverage levels. We report the estimates for assumed coverage levels of 61.6% (KEN) and 75.2% (ZAF) taken from Baral 2020 [22]. In all calculations, we used the new efficacy estimates from phase 3 clinical trials (Table 1) as inputs fitted by beta distributions (Additional file 1: SI Table 4) and duration of protection values of 3 (MV) and 5 (mAb) months.

In Kenya, we estimate that MV would reduce RSV-associated hospitalisations by 18.5% (14.5, 21.6) and RSV-associated deaths by 10.5% (7.9, 13.3). In Kenya, giving mAb to all newborns would reduce RSV-associated hospitalisations by 23.6% (20.0, 26.9) and RSV-associated deaths by 13.5% (10.7, 16.4) (Fig. 3).

In South Africa, MV would reduce RSV-associated hospitalisations by 24.2% (18.7, 28.6) and RSV-associated deaths by 27.4% (21.6, 32.3). In South Africa, giving mAb to all newborns would reduce RSV-associated hospitalisations by 33.4% (28.3, 38.0) and RSV-associated deaths by 37.9% (32.3, 43.0).

The reduction in deaths is higher in South Africa due to a stronger concentration of RSV-associated deaths in the first few months of life. Being dominated by deaths, the reduction in total disease burden will be similar to deaths. In Kenya, we estimate a reduction of total disease burden (DALYs) of 10.3% (7.7, 13) for MV and 13.2% (10.5, 16.1) for mAb. In South Africa, the reduction of total disease burden is 26.3% (20.8, 30.8) for MV and 36.5% (31.1, 41.4) for mAb.

### Cost-effectiveness

**Fig. 5:**
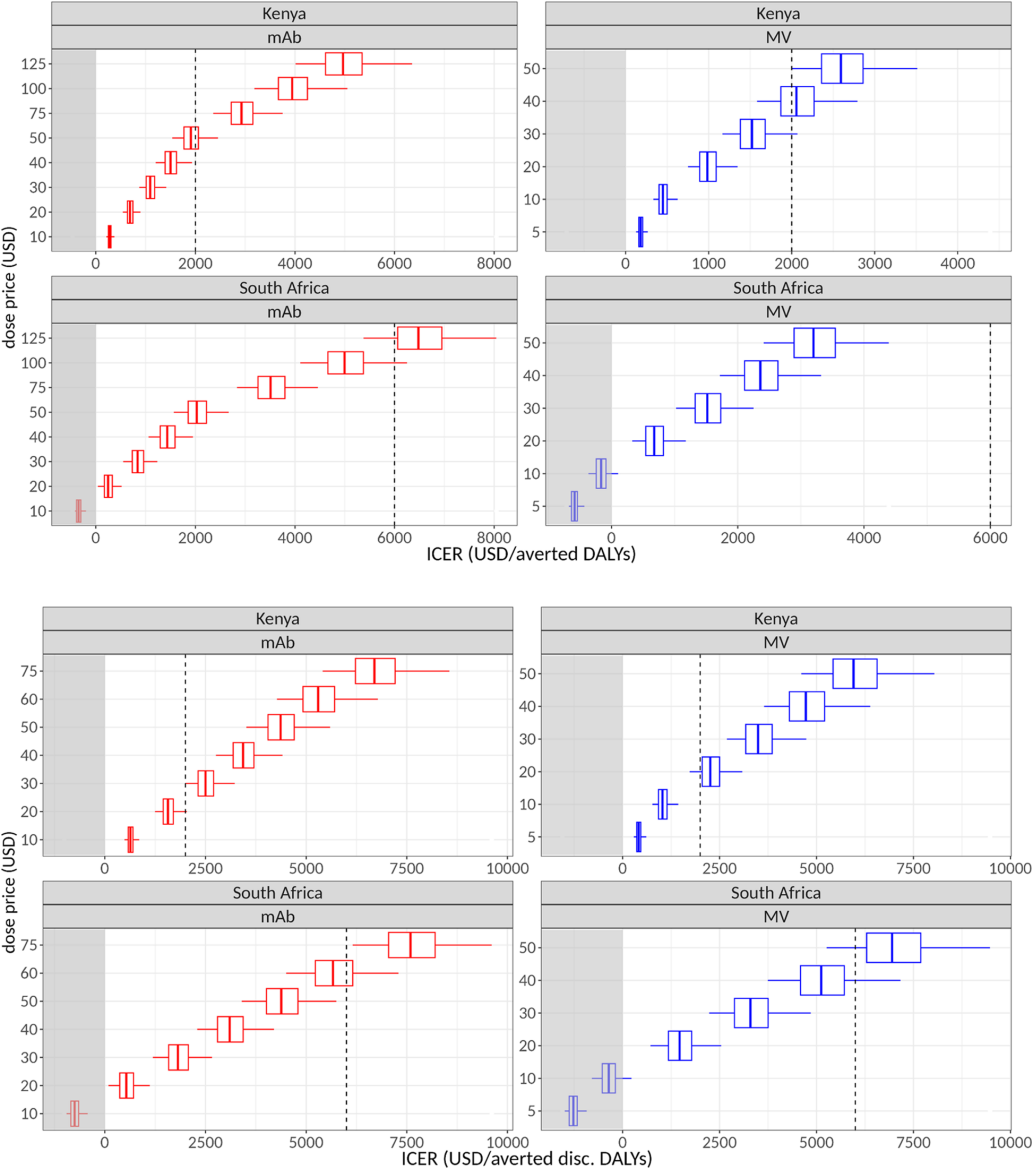
Cost-effectiveness of maternal vaccination and monoclonal antibody campaigns at different dose prices in Kenya (KEN) and South Africa (ZAF), expressed as incremental cost/DALY averted, using **A** non-discounted and **B** discounted DALYs. Negative values (area shaded in grey) represent net savings. Central lines show median values, boxes 50%, whiskers the 95% credible intervals. The dashed vertical lines show illustrative willingness-to-pay threshold values at 2000 and 6000 USD (close to the GDP/capita values of the two countries [23])

We explored a range of dose prices to see if the interventions would be cost-effective and selected a realistic price range from near-zero ICERs to a level where they exceed the GDP/capita level of the two countries (Table 2 and Fig. 5), which is approximately 2000 USD (KEN) and 6000 USD (ZAF) [23].

**Table 2:** ICER values by country and intervention type for selected dose prices. ICERs are in units of USD per averted DALYs (undiscounted)

Intervention type	Dose price (USD)	ICER, Kenya (median, 95% CI)	ICER, South Africa (median, 95% CI)
MV	5	180 (126, 267)	Cost saving (− 584 (− 714, − 421))
	10	448 (334, 627)	Cost saving (− 165 (− 366, 118))
	20	984 (750, 1349)	673 (329, 1194)
	30	1521 (1166, 2070)	1512 (1025, 2270)
	40	2057 (1582, 2791)	2350 (1720, 3346)
mAb	20	686 (543, 895)	247 (46, 510)
	40	1500 (1203, 1934)	1434 (1059, 1929)
	50	1908 (1534, 2454)	2028 (1565,2638)
	125	4964 (4011, 6351)	6481 (5364, 7959)

Using undiscounted DALYs, in Kenya, a MV with a dose price of 5 USD has an ICER value of 180 (126, 267) USD per DALY averted. The ICER is 1521 (1166, 2070) USD at a 30 USD dose price and exceeds the 2000 USD threshold at a 40 USD dose price.

For mAb in Kenya at a dose price of 20 USD, the ICER is 686 (543, 895) USD per DALY averted, and at a dose price of 50 USD, the ICER is 1908 (1534, 2454), exceeding the 2000 USD threshold above a 50 USD dose price.

In South Africa, a MV with a dose price of 5 or 10 USD has a negative ICER, meaning that it would be net cost saving. The ICER is 1512 (1025, 2270) USD at a 30 USD dose price and 2350 (1720, 3346) USD at a dose price of 40 USD, still below the 6000 USD threshold.

For mAb in South Africa at a dose price of 10 USD, the ICER is again negative and at a dose price of 50 USD the ICER is 2028 (1565, 2638), crossing the 6000 USD only at approximately 120 USD.

Using discounted DALYs (Fig. 5B), we obtain higher ICERs, exceeding 2000 USD in Kenya at a dose price of approximately 18 USD for MV and 20 USD for mAb.

For South Africa, if using discounted DALYs, the ICERs exceed 6000 USD at approximately 45 USD for MV and 60 USD for mAb.

The lower ICER values in South Africa result from a bigger reduction in the disease burden due to stronger concentration of deaths in the first few months of life, as well as larger savings for the healthcare system due to the interventions because of higher hospitalisation costs per disease episode.

### Sensitivity analysis

The calculated ICER values above are sensitive to efficacy estimates since the proportion of the disease burden averted depends on the efficacy of the MV or mAb and how that efficacy decays in time. An exponential waning model implies some level of protection lasting beyond the defined duration of protection and also leads to median ICERs lower than in the default scenarios (Additional file 1: SI Figure 2). In this case, the ICERs remain below the 2000 (KEN) and 6000 (ZAF) USD per DALY averted thresholds up to dose prices of approximately 75 (KEN) and 125 USD for MV and 100 (KEN) and 150 (ZAF) USD for mAB. This latter result strongly depends on assumptions about the half-life values (Additional file 1: SI Table 3 and Additional file 1: SI Methods 2.2).

Aggregating the incidence of deaths in 3-month age brackets only minimally changed the results; the percentage reduction in deaths due to interventions was within a 1% range of our default estimates (KEN MV: 10.5% KEN mAb: 13.6%, ZAF MV: 27.5%, ZAF mAb: 36.4%; Additional file 1: SI Figure 4). Aggregating the incidence of deaths in 6-month age brackets leads to somewhat smaller reductions of deaths (KEN MV: 8.1% KEN mAb: 12.8%, ZAF MV: 22%, ZAF mAb: 34.7%), lowering the affordable dose price by a few USD (Additional file 1: SI Figure 5). In this case, the ICER exceeds 2000 USD in KEN at a dose price of 30 USD for MV and at 50 USD for mAb; in South Africa, the ICER exceeds 6000 USD at a dose price of 70 USD for MV and at approximately 110 USD for mAB. This calculation might underestimate the cost effectiveness, as the interventions are likely to have some efficacy outside the 0–3 month (MV) and 0–5 (mAb) month windows, and additionally, the age distribution of hospitalisations is likely to be carried over to that of fatalities to some extent.

Using the narrower ILI definition for non-severe disease in South Africa does not substantially alter the estimate for total disease burden. While years lived with disability (YLD) would be lower in this case, the total burden in DALYs only changes by approximately 1% only, since it is dominated by deaths.

Herd effects have previously been estimated to be small [24]; therefore, vaccine coverage does not alter our cost effectiveness estimates, although it would lead to larger reductions in the disease burden (Additional file 1: SI Figure 3). In contrast, vaccine efficacy proportionally changes cost effectiveness (ICER) estimates as well.

## Discussion

According to our hospital surveillance data, in both Kenya and South Africa, most RSV-associated SARI cases and a large proportion of deaths occur in the first 3 months of life. For this reason, MV or mAb programmes against RSV disease may be more cost-effective than suggested by previous analyses which were based on estimates of community incidence [1–3]. Whereas in previous studies, it was assumed that severe disease and death have a similar age profile as non-severe RSV infections, our incidence data suggests severe disease is more concentrated in the first months of life, potentially increasing the impact of these interventions on DALYs and hence making them more cost-effective.

Deaths dominate the total DALY burden of RSV disease for infants. Phase 3 data from recent trials showed efficacy of 81.8% (40.6, 96.3) against severe MA-LRTI for the MV RSVpreF and 77.3% (50.3, 89.7) for the mAb candidate nirsevimab (Beyfortus), which would lead to significant reductions in deaths.

Since most of the total disease burden measured in DALYs (Fig. 3b) is due to deaths, the main driver of cost-effectiveness of the intervention is its impact on deaths. If coverage levels of 61.6% (KEN) and 75.2% (ZAF) can be achieved [22], these efficacy figures could lead to a reduction in deaths of 11% (MV) to 14% (mAb) in Kenya and 27% (MV) to 38% (mAb) in South Africa and an almost equivalent reduction in total disease burden.

Additionally, since medical costs are dominated by hospitalisations associated with SARIs, the comparable reduction in hospitalisations leads to a similar reduction in RSV-associated medical costs. As a combined effect of these two, we arrive at median estimates of (undiscounted) ICERs below 2000 USD in Kenya for a MV with a dose price up to 40 USD and for mAb with a dose price up to 50 USD (Fig. 5).

In South Africa, with its higher hospitalisation costs, the ICERs appear to be even more favourable. If the willingness-to-pay is 6000 USD, a MV costing 30 or 40 USD per dose is well below this level (Table 2), whereas for mAb, the ICER reaches 6000 at approximately 120 USD. In South Africa for dose prices at or below 10 USD for MV, the intervention may be net cost saving, while for mAb, it is cost saving up to approximately 15 USD (Fig. 5).

The mAb nirsevimab (Beyfortus) was recently granted approval by the European Commission [25] and the MHRA (UK) [26]. The MV candidate RSVpreF (PF-06928316) was accepted for priority review by the FDA with a goal date of May 2023 for a decision on approval [27]. Therefore, these two products might enter large-scale use in the next 2 years. There are several other vaccine candidates in different phases of clinical trials, mostly paediatric or older-adult vaccines [6, 28], which however could also affect the RSV burden for infants through herd effects.

Our study has several limitations. The estimates on disease burden reduction and cost-effectiveness are sensitive to the confidence intervals of the efficacy data of the MV [20] and mAb [8, 9, 21] and to the probability distributions used to fit them. In the case of MV, we used phase 3 data made public by the drug manufacturer but not yet peer-reviewed. We expect that with the full results becoming available confidence intervals of efficacy estimates might become less skewed and narrower, enabling more accurate estimates. The latest efficacy results for MV indicate [20] that it provides some protection in the age group from 3 to 6 months as well. Since the results were not disaggregated by age, we could not estimate this effect separately and instead conservatively assumed an effect only in the 0 to 3 months age group in the default case, while there is some protection in the exponential decay scenario, extrapolated from the efficacy for the 0 to 3 months age group. If the MV provides a level of protection from 3 to 6 months of age as well, this will further reduce the disease burden and improve the cost effectiveness of MV.

We did not consider a scenario where interventions are given before and during RSV seasons rather than throughout the whole year. Seasonal immunisation campaigns are likely to be more efficient (per dose) [29] in countries with a clear seasonal RSV pattern. However, on the national level, Kenya does not have a clear RSV season pattern [30], although some of its regions do, but even these regions have more spread-out RSV circulation than temperate countries [31]. Therefore, it is not clear if seasonal campaigns would be viable as opposed to nationwide year-round immunisation. A seasonal approach could be more viable in South Africa as it has a more seasonal pattern nationally [31, 32] although with substantial variation in the timing of the season’s end. Contact restrictions during the COVID-19 pandemic disrupted RSV seasonality in South Africa [33], adding further uncertainty to the timing of RSV seasons in the near future. Our model assumed that intervention costs are proportional with coverage and therefore the coverage level does not change the cost-effectiveness of interventions. In reality, part of the costs required to introduce and implement the interventions are likely to be fixed and do not vary with the number of doses administered [34], and therefore, a higher coverage can further improve the cost effectiveness (lower the ICER).

The concentration of SARI illness episodes observed in children under 6 and in particular under 3 months of age in Kenya and South Africa appears to be different from the age distribution observed in some other African countries [17]. Some incidence rates for regions in Kenya had to be projected from the areas of data collection using data on health-seeking behaviour. More sites for data collection would further support the reliability of these estimates. Further studies would be important to investigate if this concentration of severe RSV disease in early infancy is a more general phenomenon in African countries or perhaps specific to the two countries analysed in the current study. Moreover, the estimates on deaths were derived by combining the hospitalisation data with estimates of CFR values and the proportion of out-of-hospital deaths. Our estimates of the CFR values and proportion of out-of-hospital deaths only had 3- or 6-month resolution in the under-1 year age groups and large age groups for the above-1 year olds. Studies measuring the incidence of deaths with a finer resolution would be required especially in the 3- to 9-month range where we report high CFR values and out-of-hospital deaths. In these age brackets, the monthly distribution of deaths could make a large difference because they might be at the limit of the time window where MV and mAb are efficacious. Indeed, there is still uncertainty about the duration of efficacy of MV as results have not yet been fully published. If the duration of protection extends into the period of 3 to 6 months after birth, this will improve the cost effectiveness further.

Out-of-hospital deaths in Kenya were estimated from the hospitalised SARI burden and the ratio of out-of- to in-hospital deaths from multiple LMICs [5], resulting in a high number of out-of-hospital deaths in the first months of life and in age 6 to 11 months. These out-of-hospital deaths make the interventions in Kenya more cost-effective, although this is counterbalanced by the deaths outside the first 6 months of life left unaffected by the interventions, assuming that the efficacy falls to zero after 3 (MV) or 6 (mAb) months.

## Conclusions

In summary, our results suggest that public health interventions based on vaccination or monoclonal antibodies against RSV disease in infants in two African countries might be cost-effective, depending on the country’s willingness-to-pay values. Specifically, in Kenya, dose prices up to 40 USD for MV and up to 50 USD for mAb result in ICER values below the country’s GDP per capita level (we used 2000 USD above). In South Africa, a dose price of 40 USD for MV and 120 USD for mAb results in ICER values below the country’s GDP per capita (we used 6000 USD above), while dose prices below 10 USD (MV) and 15 USD (mAb) might be net cost saving.

As recent estimates of cost-effectiveness thresholds based on health opportunity costs [19] tend to be below previously commonly used thresholds in the range of 1–3× of GDP per capita, the newest efficacy figures suggest interventions against RSV disease by these preventive biologics may be more widely viable, if priced in an affordable range. In the coming years, further analysis and planning for these interventions in the light of final efficacy estimates from phase 3 clinical trials will continue to be an important priority for public health policy.

## Supplementary Information

**Additional file 1: SI Fig 1.** Cost of RSV-associated hospitalisation in South Africa by age group. **SI Fig 2.** Incremental cost per DALY averted when assuming exponentially waning efficacy. **SI Fig 3.** Reduction in disease burden and costs, assuming a higher coverage level equivalent to BCG coverage. **SI Fig 4.** Reduction in disease burden and costs, if merging death incidence estimates in 3-month age brackets. **SI Fig 5.** Reduction in disease burden and costs, if merging death incidence estimates in 6-month age brackets. **SI Table 1.** Data sources and definitions for metrics used. **SI Table 2.** Parameter values used in the analysis. **SI Table 3.** Parameter estimates for exponentially waning efficacy. **SI Table 4.** Parameter estimates for fits of efficacy data with beta distributions. Supplementary Methods: additional information on data availability, data collection methods and fitting the efficacy data [35–56].

## Data Availability

Computer scripts used for the cost-effectiveness analysis and the corresponding data input files are available at https://github.com/mbkoltai/RSV-CEA-Kenya-South-Africa. We provide more detailed information on data availability in Additional file 1: Supplementary Methods.

## Abbreviations

DHS: Demographic and Health Surveys
HCW: Healthcare worker
HMS: Household Morbidity Surveillance
HUS: Healthcare utilisation survey
LRTI: Lower respiratory tract infection
mAb: Monoclonal antibodies
MV: Maternal vaccination
