# Supplementary material for: Estimating the cost-effectiveness of maternal vaccination and monoclonal antibodies for respiratory syncytial virus in Kenya and South Africa
Source: BMC Med. 2023 Mar 31;21:120. doi: 10.1186/s12916-023-02806-w (PMC10064962; doi:10.1186/s12916-023-02806-w)
Supplement: Supplementary file 1 — Additional file 1: SI Fig 1. Cost of RSV-associated hospitalisation in South Africa by age group. SI Fig 2. Incremental cost per DALY averted when assuming exponentially waning efficacy. SI Fig 3. Reduction in disease burden and costs, assuming a higher coverage level equivalent to BCG coverage. SI Fig 4. Reduction in disease burden and costs, if merging death incidence estimates in 3-month age brackets. SI Fig 5. Reduction in disease burden and costs, if merging death incidence estimates in 6-month age brackets. SI Table 1. Data sources and definitions for metrics used. SI Table 2. Parameter values used in the analysis. SI Table 3. Parameter estimates for exponentially waning efficacy. SI Table 4. Parameter estimates for fits of efficacy data with beta distributions. Supplementary Methods: additional information on data availability, data collection methods and fitting the efficacy data [35–56]. [file 12916_2023_2806_MOESM1_ESM.docx]

# Supplementary material to “*Estimating the cost-effectiveness of maternal vaccination and monoclonal antibodies for respiratory syncytial virus in Kenya and South Africa from hospital and community incidence data*”

## SI Figures


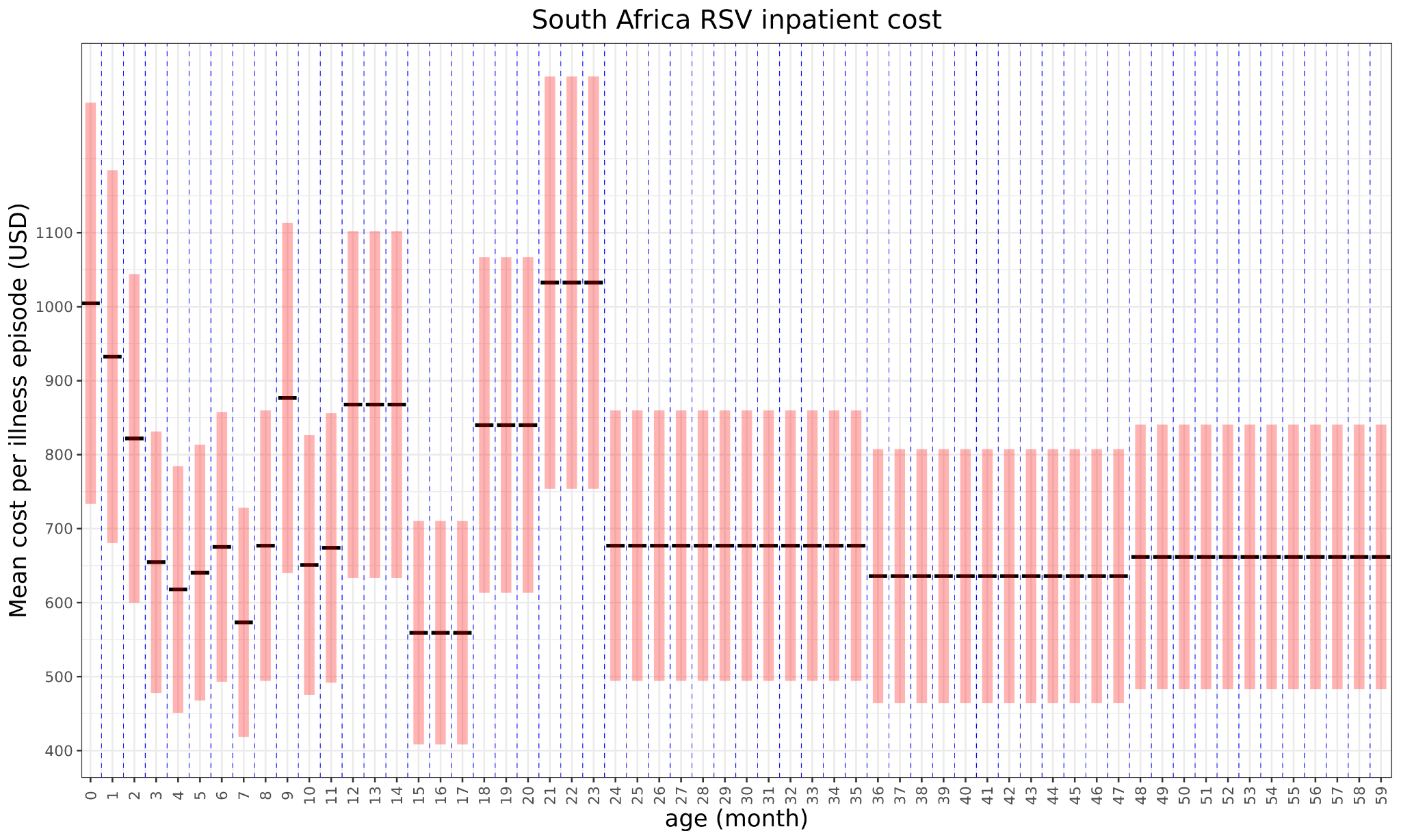


**SI Figure 1**: Cost of RSV-associated hospitalisation in South Africa by age group (mean and 95% confidence intervals). The variation above 5 months of age is mainly due to whether there were ICU cases in a given age band. Since most hospitalisations are in the first months of life this variation in the older age groups does not significantly impact the results.


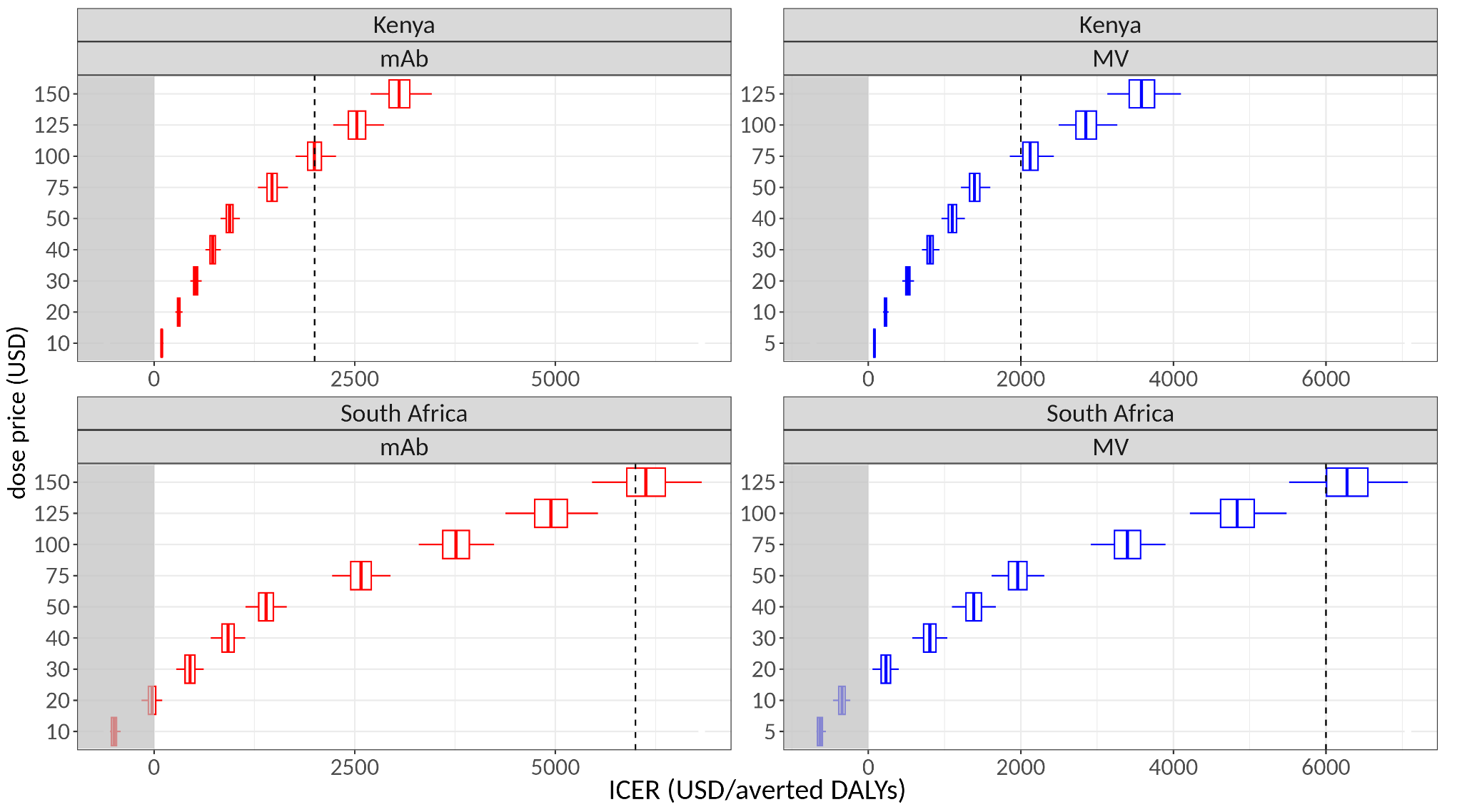


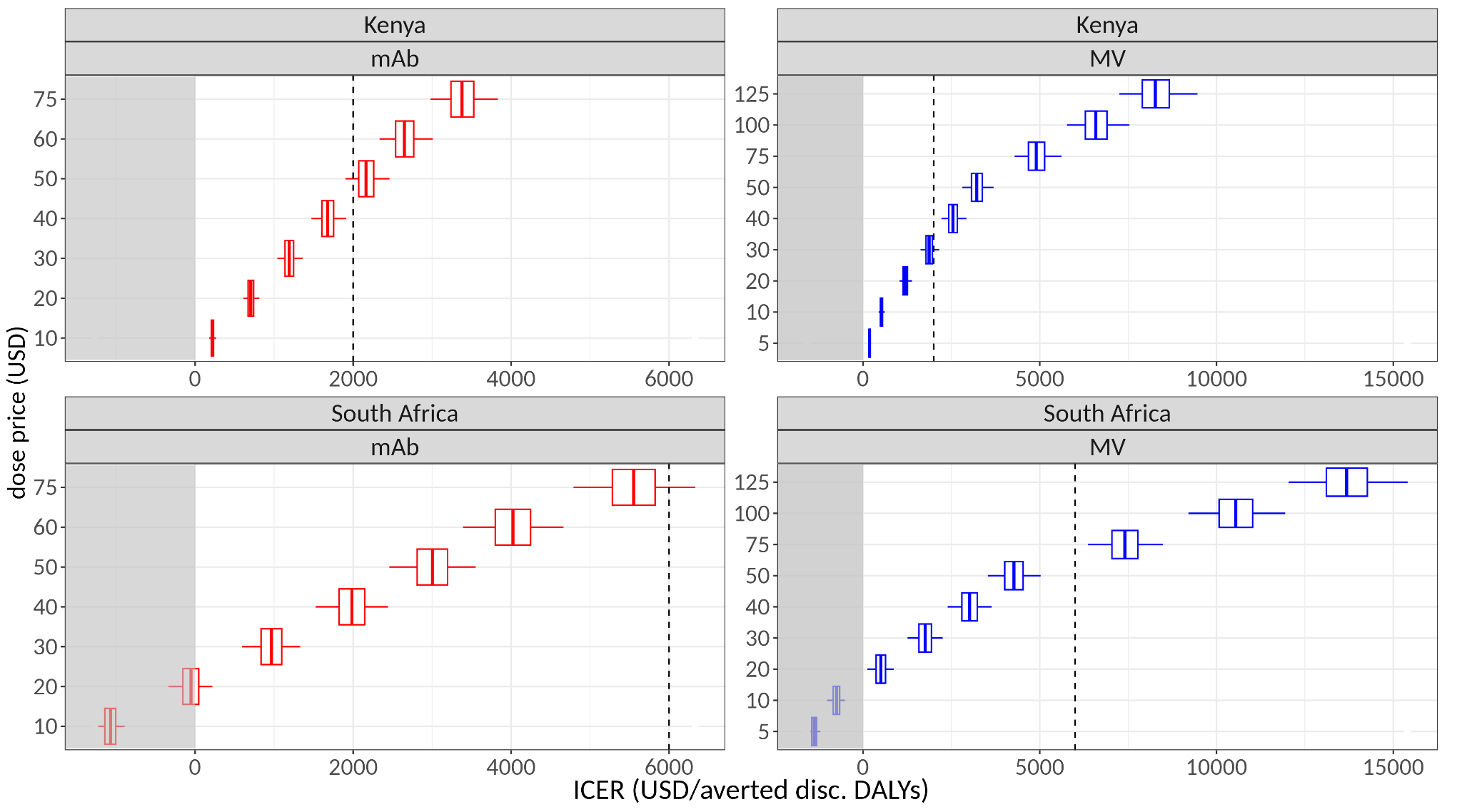


**SI Figure 2**. Incremental cost per DALY averted when assuming exponentially waning efficacy instead of a step function. Efficacy figures and parameters for exponential waning are in SI Table 2 and 3. Upper plot shows undiscounted, lower plot discounted DALYs.


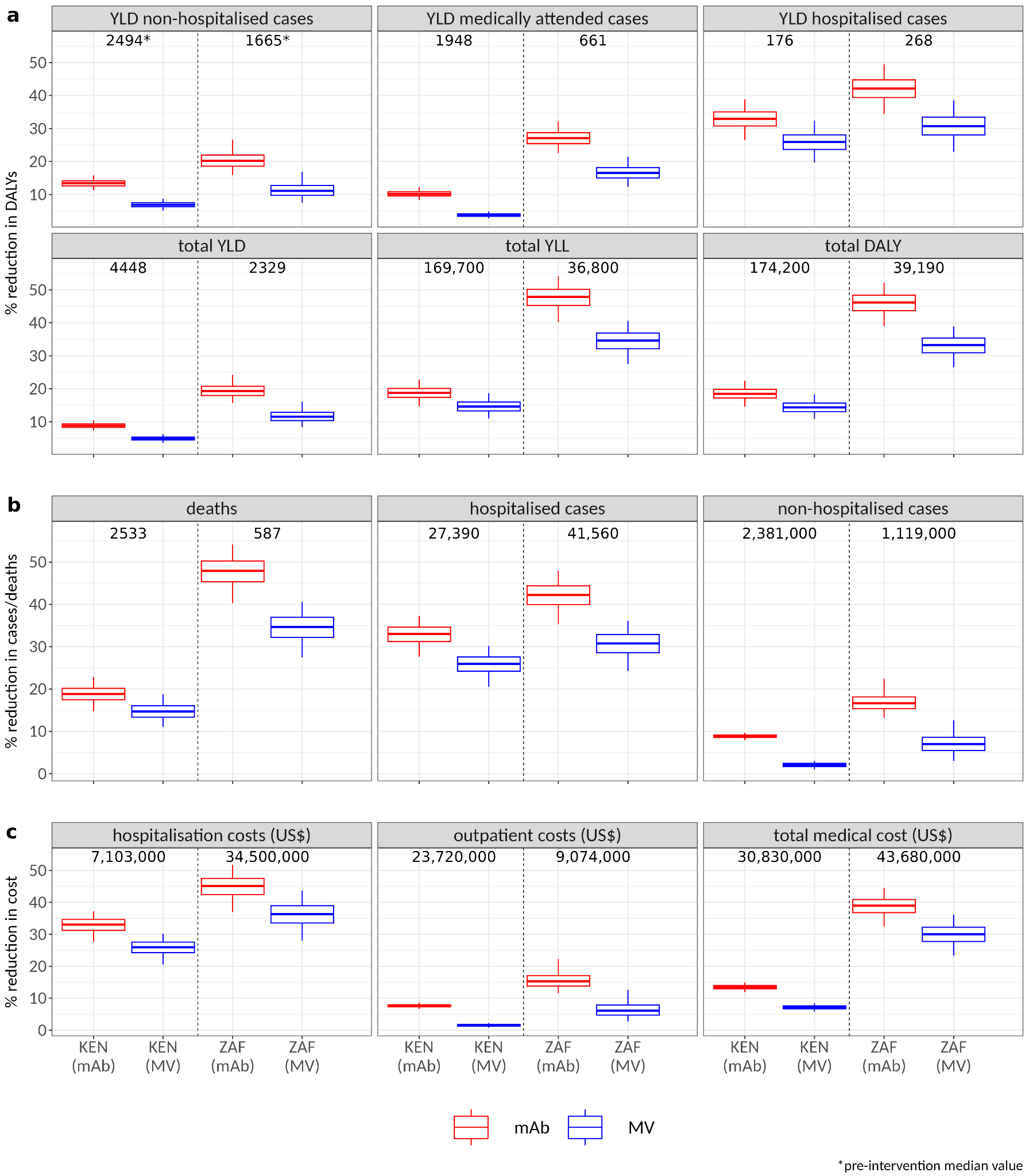


**SI Figure 3** Reduction in disease burden and costs, assuming a higher coverage level equivalent to BCG coverage (KEN: 86%, ZAF: 95%) [12]. Due to the higher coverage level, the reduction in deaths is approximately 1.3-1.4x higher than in the case of using the inferred coverage level based on ANC-3 data [20], which was done in the main text.


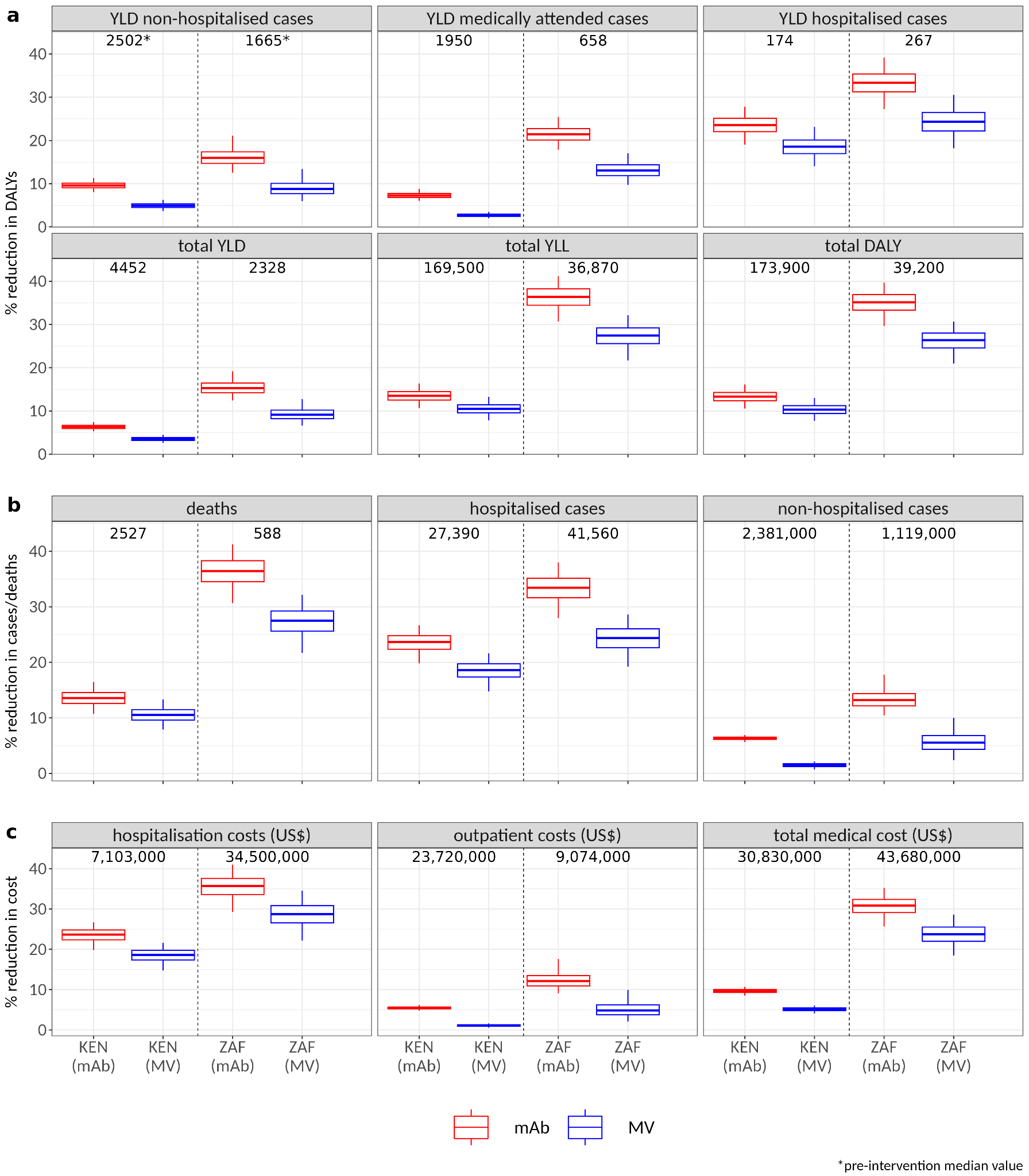


**SI Figure 4** Reduction in disease burden and costs, if merging death incidence estimates in 3-month age brackets.


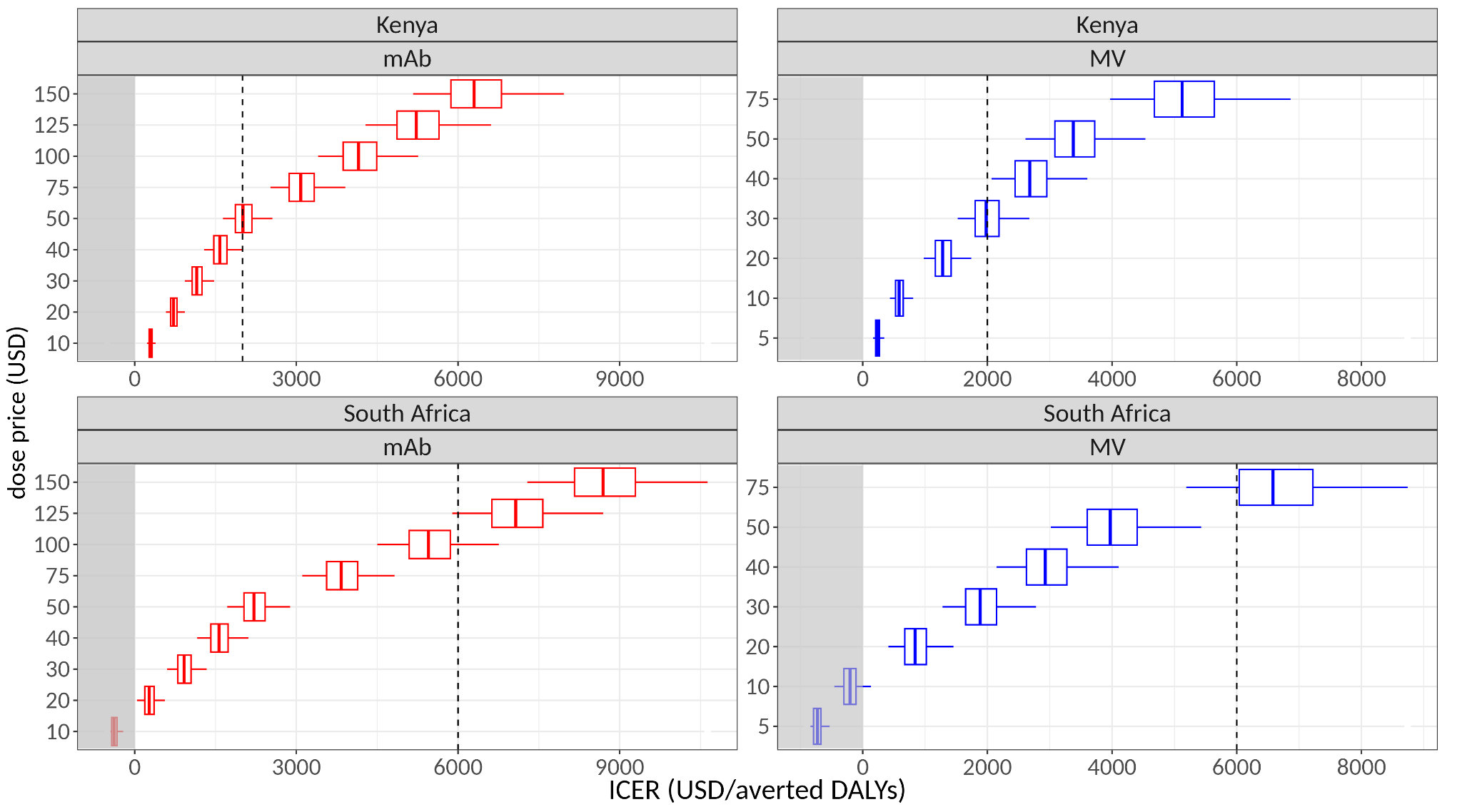


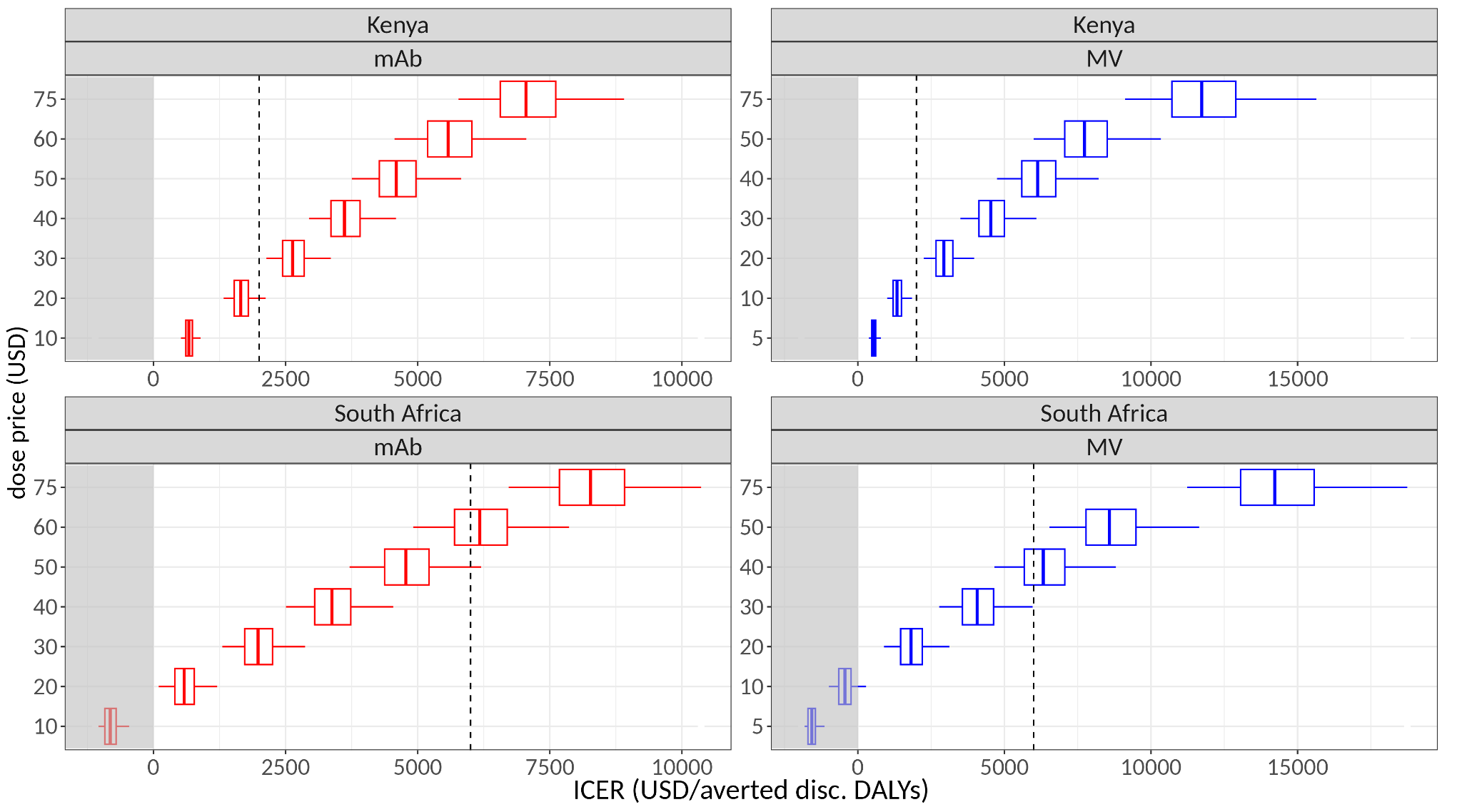


**SI Figure 5** Reduction in disease burden and costs, if merging death incidence estimates in 6-month age brackets.

## **SI Tables**

##

## **SI Table 1**: Data sources and definitions for metrics used

| Metric | Disease type | Source, country | Type of data | Calculation |
| --- | --- | --- | --- | --- |
| I_A_ = Rate of medically attended ARI in Asembo (base area) | ARI: acute respiratory infection.  Definition: acute respiratory infection (onset within 10 days) with cough, difficulty breathing, sore throat or runny nose | Kenya Medical Research Institute (KEMRI),  KENYA | Hospital data from St. Elizabeth Lwak Mission Hospital (LMH) and surveillance data from Health and Demographic Surveillance System (HDSS) in Asembo, Kenya | 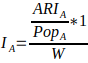  ARI_A_ = Total number of ARI cases in Asembo who visited LMH per year  Pop_A_ = Population of surveillance catchment area. The catchment area is defined in [34]. There was ongoing surveillance, so population and healthcare-seeking behaviour data are up-to-date.  W = Proportion of ARI cases visiting LMH *among those who sought care*, from Household Morbidity Surveillance (HMS) [35] |
| I_B_ = Rate of medically attended ARI in the base region | ARI | KEMRI,  KENYA | Projected from rate of medically attended ARIs in Asembo by :  - health seeking behavior of ARI cases in Asembo from Household Morbidity Surveillance (from HMS)  - Proportion of ARI cases seeking care in Nyanza region (from DHS) | 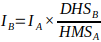  DHS_B_ = Proportion of ARI cases seeking care in base region from DHS [21]  HMS_A_ = Proportion of ARI cases seeking care, in Asembo (from HMS [35])  DHS: Demographic and Health Survey 2015 [36] |
| I_M,Y_ = Incidence of medically attended and non-attended ARI in region Y  I_NM,Y_ = Incidence of non-medically attended ARI in region Y | ARI | KEMRI,  KENYA | ARI rate for base region (Nyanza) projected by adjustment with risk factors [36,37] | 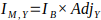  Adj_Y_ = adjustment factor.  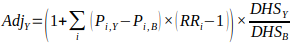  P_i,Y_ =prevalence of risk factor i in region Y  P_i,B_ =prevalence of risk factor *i* in the base region (Nyanza province).  Risk prevalence estimated from [36,37]. 1000 iterations were run for each risk factor (binomial distribution) to estimate credible intervals.  RR_i_=Relative risk of ARI due to risk factor *i*, from [22]  DHS_Y_ = Proportion of ARI cases seeking care in region Y (from DHS)  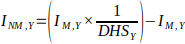 |
| IF_M,Y_=Incidence of medically attended RSV-associated ARI in region Y  IF_NM,Y_ = Incidence of non-medically attended RSV-associated ARI in region Y | ARI | KEMRI,  KENYA | Calculated from projected ARI rate for region Y:  - ARI and SARI RSV testing data of base region: used for *Nyanza region*  - testing data from Siaya County Referral Hospital (CRH) and Kakamega CRH used for *Western Province*  - Kenyatta National hospital (NH), Nakuru CRH, Nyeri CRH used for *Rift valley, Central and Nairobi regions*  - national average including testing from Marsabit CRH and Coast General Teaching and Referral Hospital (GTRH) used for *Eastern and North Eastern regions* | 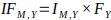  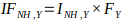  F_Y_ = Proportion of ARI due to RSV in region Y  Nasopharyngeal and Oropharyngeal (NPOP) samples were systematically tested in Asembo for all enrolled cases in the study. This provided the RSV positivity for the base (Nyanza) region.  The ratio of RSV-positivity for ARI and SARI in the base region was applied to the SARI testing data of other regions to get the RSV-positivity for ARIs in the other regions.  The percent of ARIs positive for RSV in the base region was aggregated for the entire period with data (2010-14) and grouped into 3-month age bands from <3 months of age to 23 months, and 12-month age bands from 24 months of age. |
| I_B_ = Annual base rate of hospitalised SARI | SARI  Definition: acute respiratory infection (onset within 10 days) with cough and reported or documented fever of ≥38C. For non-hospitalised severe cases, pneumonia was used as a proxy for SARI.  For non-hospitalised SARIs pneumonia was used as a proxy for healthcare seeking behaviour. Pneumonia was defined as a cough and difficulty breathing for >2 days or a diagnosis of pneumonia by a HCW (within the last year, when identified retrospectively). | KEMRI,  KENYA | Data collected from children <5 years visiting Kilifi CRH and residency data from participants in the Kilifi *Health and Demographic Surveillance System* (HDSS) [38]. | I_B_ = SARI_B_/POP_B_  I_B_ was calculated for participants of the Kilifi HDSS for the years 2010-18 by dividing the age-specific number of hospitalised SARI by the age-specific population of HDSS residents in Kilifi.  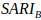 = Total number of cases meeting SARI case definition hospitalised in surveillance catchment area  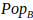=Population of surveillance catchment area |
| I_H,Y_ = Incidence of hospitalised SARI in region Y | SARI | KEMRI,  KENYA | Projecting the SARI rate of base region by using:  - regional data for healthcare seeking behaviour from KHDSS  - regional data for risk factors | 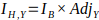  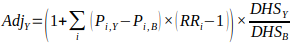  Risk factor prevalence DHS_B_ and DHS_Y_ from KHDSS. Risk factor contribution to SARI from [37].  Relative risk of HIV estimated 3.1% (95% CI, 1.2–8.1) from [39].  Credible intervals calculated from 1000 iterations allowing each risk factor to vary within a binomial distribution defined by the proportion with the characteristic and numbers interviewed. |
| I_NH,Y_ = Incidence of non-hospitalized SARI in region Y | SARI | KEMRI,  KENYA | From I_NH,Y_ by using data on healthcare seeking for acute respiratory illness from the 2018 Health Utilization Survey (HUS) data (unpublished) conducted in four counties (Siaya [Nyanza], Marsabit [Eastern], Nakuru [Rift Valley], Kakamega [Western]).  Proportion of individuals hospitalised with pneumonia was used as a proxy for SARI hospitalisation. Pneumonia definition used was: cough AND difficulty breathing >2 days OR a diagnosis by a HCW within the last year.  For Nyanza, Eastern, Rift Valley and Western regions the HUS estimates were used.  For Central, Nairobi and North Eastern the HUS estimates are adjusted by the care-seeking behaviour of these regions compared to national average from KHDSS. | 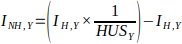  HUS_Y_ = proportion of all SARI cases hospitalised in region *Y*.  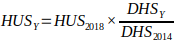  HUS_2018_ = Proportion of all hospitalised SARI cases in the four counties  DHS_Y_ = Proportion of ARI cases seeking care in region Y (from KDHS).  DHS_2014_ = Proportion of ARI cases seeking care nationally (from KDHS)  Using the DHS_Y_/DHS_2014_ ratios assumes that healthcare seeking for SARIs varies by region proportionally to healthcare seeking in the case of ARIs. |
| Incidence of hospitalized/non-hospitalised RSV-associated SARI in region Y | SARI | KEMRI,  KENYA | Rates of hospitalised/ non-hospitalised SARI (I_H,Y_, I_NH,Y_) estimated as described above.  RSV positivity estimates:  - For Coastal and Nyanza regions: RSV positivity is from systematic testing of NPOP samples from hospitalised SARI patients.  For other regions testing was intermittent.  - Western region: Siaya CRH testing data was combined with Kakamega CRH.  - Rift Valley, Central and Nairobi regions: data from Kenyatta National hospital (NH), Nakuru CRH and Nyeri CRH were combined.  - Eastern and North Eastern regions: the national average was used combining the above data sources with Coast GTRH. | 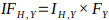  F_Y_ = Proportion of SARIs testing positive for RSV  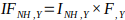 |
| I_B_ZA,i_ : Incidence of hospitalised SARI cases per population in base provinces, for age group *i* | SARI definition: children admitted to hospital with a physician’s diagnosis of LRTI, with or without fever and a duration of 10 days or less. | National Institute For Communicable Diseases Of South Africa (NICD),  SOUTH AFRICA | Two data sources described in [14]:  1) Hospital surveillance: RSV surveillance among patients with ILI, SARI and SARI in 3 public hospitals in 2 provinces, population of 457,000 (1.3% of South Africa) in 2015.  Sites are:  - Edendale Hospital (KwaZulu-Natal)  - Klerksdorp and Tshepong Hospitals (North-West)  Respiratory specimens collected from all enrolled patients (ILI, SARI, SARI) and tested for 10 different viruses, among them RSV.  2) Healthcare utilization surveys: from studies of healthcare seeking behaviour among individuals with reported ILI or SARI in three South African communities. | I_B_ZAF,i_ = [SARI_enrolled_*(7/5)*(1/X_SARI_i_)*(1/HUS_SARI_)]/Pop_i_  SARI_enrolled_: age-specific number of SARI cases enrolled  The factor 7/5 is used to adjust for non-enrolment over weekends.  X_SARI_i_: proportion of all enrolled SARI cases belonging to age group *i*  HUS_SARI_: proportion of SARI cases that sought care at the surveillance site out of SARI cases that sought care in any hospital obtained from the HUSs conducted in the catchment area of surveillance site [40,41]. |
| I_H,Y_: Incidence of hospitalised SARI cases per population in other provinces, for age group *i* | SARI | NICD,  SOUTH AFRICA | Risk factors of pneumonia for the other 7 provinces were taken from the DHS [42].  These included HIV infection, exposure to indoor air pollution, crowding, malnutrition, low birth-weight and non-exclusive breastfeeding (last 3 only for children <5yr). SARI-association of risks from [43,44].  Provincial rates were also adjusted by proportion of ARI cases seeking care in the given province relative to the base provinces [45,46], ie. healthcare-seeking among ARIs was used as a proxy for SARIs. | I_H,Y_ = I_H,B_ * Adj_Y_ * DHS_H,Y_/DHS_H,B_  Adj_Y_=$1+\sum_{i=1}^{n} \left( P_{i,Y}-P_{i,B} \right)\left( {RR}_{i}-1 \right)$  DHS_H,Y_: proportion of ARI cases seeking care in province Y (from DHS [18], [28], [29])  DHS_H,B_: proportion of ARI cases seeking care in the base provinces |
| IF_H,Y_ZAF_: RSV-associated SARI hospitalisation in all provinces | SARI | NICD,  SOUTH AFRICA | Testing procedure described in [35]. Respiratory specimens were collected from all enrolled patients, and were tested for the presence of 10 respiratory viruses (including RSV) using a multiplex real-time reverse-transcription PCR assay. | IF_H,Y_ZAF_= I_H,Y_*F_Y_*F_Y,AF_  I_H,Y_: SARI hospitalisation rate in province Y (including the base provinces)  F_Y_: proportion of SARI cases testing positive for RSV [26]  F_Y,AF_: attributable fraction of RSV virus detection to illness, obtained at the same sentinel sites [47] |
| I_NH,B_ZAF_: Incidence of non-medically-attended SARI cases per population in base provinces | SARI | NICD,  SOUTH AFRICA | From HUS data [40,41] | I_NH,B_ZAF_=I_H,B_ZAF_*(1/HUS_B_ - 1)  HUS_B_: proportion of all SARI cases that are hospitalized in the base provinces |
| I_NH,Y_ZAF_ : Incidence of non-medically-attended SARI cases per population in other provinces | SARI | NICD,  SOUTH AFRICA | From HUS data and estimates on risk factors | I_NH,Y_ZAF_=I_NH,B_ZAF_*Adj_Y_ * DHS_NH,Y_/DHS_NH,B_  (all variables already defined above) |
| IF_H,Y_ZAF_: RSV-associated non-hospitalised SARI rate in all provinces | SARI | NICD,  SOUTH AFRICA | From previous variables and RSV testing data at sentinel sites | IF_NH,Y_ZAF_= I_NH,Y_*F_Y_*F_Y,AF_  I_NH,Y_: SARI hospitalisation rate in province Y (including the base provinces)  F_Y_: proportion of SARI cases testing positive for RSV  F_Y,AF_: attributable fraction of RSV virus detection to illness, obtained at the same sentinel sites [48] |
| ILI_i_: age-specific rate of RSV-associated ILI outpatient consultations in age group *i* | Definition: outpatient of any age presenting with either temperature ≥38C or history of fever and cough of a duration ≤10 days.  We approximated ARIs from ILIs by taking the proportion of ARIs with fever (on average 23.3% from 2015 to 2017) in Kenya [49] and dividing the ILI rates by this proportion. | NICD,  SOUTH AFRICA | From SARI rates, combined with proportion of ILI consultations referred to hospital and proportion of SARI cases that previously seeked outpatient care | ILI_i_ = SARI_i_ * Z * X * (Y_ILI_i_ / Y_SARI_i_)  SARI_i_: age specific rate of SARI hospitalization (adjusted for non-enrollment and healthcare seeking behavior)  Z: proportion of SARI cases that sought outpatient care before hospitalization  X: ratio of ILI consultation referred to hospital to the total number of ILI consultations obtained from ILI surveillance over the study period  Y_ILI_i_: proportion of ILI cases in age group *i* over the total number of ILI cases after adjusting for non-enrolment  Y_SARI_i_: proportion of SARI cases in age group *i* over the total number of SARI cases after adjusting for non-enrolment |

**SI Table 2**: Parameter values used in the analysis

| Parameter | Type | value | Source |
| --- | --- | --- | --- |
| Duration of protection | Maternal vaccination | Default case: 3 months of protection with efficacy figures above.  With exponential waning vaccine efficacy is a function of time; parameters in SI Table 3. | [18] |
| Duration of prot. | Monoclonal antibody | Default case: 5 months protection with efficacy figures above, 0% protection after.  With exponential waning vaccine efficacy is a function of time; parameters in SI Table 3.  See SI Methods for calculation. | [18] |
| Cost | Hospitalisation, South Africa | See SI Figure 1, between 559 and 1032 USD per illness episode | own data |
| Cost | Outpatient care, South Africa | 25 USD (CI95: 18.3, 31.8) | own data |
| Cost | Outpatient care,  Kenya (Siaya) | 20.91 USD (calculated from total cost to the hospital at Siaya, total number of inpatient and outpatient cases, and an assumed ratio of inpatient to outpatient costs of 4.9) | own data |
| Cost | Inpatient care,  Kenya | 102.46 USD (see above) | own data |
| Cost | Costs incurred to households prior to hospitalisation, Kenya (Siaya) | mean: 34.67USD (median: 14.23, CI95 12.86-56.49) | own data |
| Cost | Costs incurred to households during hospitalisation, Kenya (Siaya) | Mean: 138.51USD (median: 122.7, CI95: 106.54-170.48) | own data |
| Cost | Costs incurred after hospitalisation, Kenya (Siaya) | 15.35USD (median: 4.61, CI95: 0-34.46) | own data |
| Cost | Number of inpatients in 12 months, Kenya (Siaya) | 7330 | own data |
| Cost | Number of outpatients in 12 months, Kenya (Siaya) | 62604 | own data |
| Cost | Total healthcare cost in 12 months | 2,060,208 USD | own data |
| Cost | Maternal vaccination | 3, 10, 30 USD per dose | own data |
| Cost | Monoclonal antibody | 6, 20, 60 USD per dose | own data |

**SI Table 3**: parameter estimates for exponentially waning efficacy (fitting efficacy figures in Table 1, same sources, fitting method described in SI Methods 2.2)

| **Intervention** | **Endpoint** | ***c*** | ***α* (in months)** |
| --- | --- | --- | --- |
| MV | Medically attended LRTI | 1 | 0.738 |
| MV | Severe MA-LRTI (first 90 days life) | 1 | 0.214 |
| mAb | RSV MA-LRTI (first 151 days of life) | 1 | 0.123 |
| mAb | RSV LRTI hospitalisation | 1 | 0.139 |

**SI Table 4**: Parameter estimates for fits of efficacy data with beta distributions. Fits were performed by the *optim* function in R [50]. The exact numerical values of fitting parameters might vary with individual fits (fits to efficacy figures in SI Table 2, sources are the same)

| **Drug** | **Disease type** | **Empirical mean** (CI95),  **Fitted mean** (CI95) | **Fitted beta distribution parameters** (α,β)  (rounded to 4 meaningful digits) |
| --- | --- | --- | --- |
| Pfizer MV candidate | Medically attended LRTI | 57.1% (14.7,79.8) [18]  fit: 57.1 (15.3 , 92.8) | α=2.630, β=1.976 |
| Pfizer MV candidate | Severe MA-LRTI (first 90 days life) | 81.8% (40.6, 96.3) [ibid]  fit: 81.8 (50.7, 98.7) | 6.31, 1.404 |
| mAb Nirsevimab (Beyfortus) | RSV MA-LRTI (first 151 days of life) | 79.5% (65.9, 87.7) [19]  fit: 79.5 (65.9, 90.4) | 31.623, 8.154 |
| mAb Nirsevimab (Beyfortus) | RSV LRTI hospitalisation | 77.3% (50.3, 89.7) [ibid]  fit: 77.3 (50.5, 95.2) | 9.120, 2.678 |

##

##

## Supplementary Methods

1. Data: methods and definitions

1.1 Incidence data from Kenya: methods and definitions

1.2 Incidence data from South Africa: methods and definitions

1.3 Data availability

1. Fitting efficacy data

2.1 Fitting confidence intervals of efficacy estimates by beta distributions

2.2 Exponential waning model for efficacy figures of MV and mAb

1. Comparison with parameter estimates in Li et al

**1.1 Incidence data from Kenya: methods and definitions**

To estimate the RSV-associated ARI burden in Kenya, methods previously used for estimating the burden of outpatient ILI and RSV-associated ILI in Western Kenya [51] were combined with those used in estimating the burden of SARI and influenza-associated SARI [39,52].

ARI was defined as an acute (onset within 10 days before diagnosis) illness with cough, difficulty breathing, sore throat or runny nose. The definition for extended SARI, in line with the WHO’s RSV surveillance case definition [53], was hospitalisation with an acute respiratory infection (onset within 10 days) with cough or difficulty breathing. For non-hospitalised severe cases, ARI with pneumonia was used as a proxy for SARI. The definition for pneumonia was a cough and difficulty breathing for >2 days or a diagnosis of pneumonia by a health care worker (HCW) within the last year.

First, in order to estimate RSV-associated ARI, the overall ARI rate in Kenya was established. To do this, data on healthcare-seeking behaviour from households reporting ARI in the Household Morbidity Surveillance (HMS) [35] were used to estimate the proportion of ARI cases seeking formal medical care, by collecting data from over 27 thousand participants at biweekly home visits and at Lwak Hospital (rural western Kenya) where free care was provided by dedicated study clinical staff, asking ill persons if and where they sought care. The total number of ARI cases per year were collected from households in the base area of Asembo (Siaya County, Nyanza Region) in the catchment area of St. Elizabeth Lwak Mission Hospital (LMH) and through the Population-Based Infectious Disease Surveillance system (PBIDS) [54]. In the latter study field workers visited households in rural western Kenya (Asembo) and an informal settlement in Nairobi every two weeks collecting recent illness information; participants (>50,000 persons) could access free high-quality care in a referral clinic at each site. The ARI rate in the base area was calculated by dividing the number of ARI cases visiting LMH in a year by the population of the surveillance catchment area, scaled by the proportion of ARI patients who seek care from HMS. This rate was projected to the larger base region (Nyanza) by the ratio of ARI cases seeking care in the region [36] to those seeking care in Asembo [35]. Risk factors for respiratory infections (such as air pollution, malnutrition, or HIV prevalence) [37] were used in a linear model to project the ARI rate to other regions, while also accounting for different regional levels of healthcare-seeking behaviour. Data on healthcare-seeking behaviour was from a Health Utilisation Survey (HUS; unpublished data) conducted in Siaya County (Nyanza Region), Nakuru County (Rift Valley), Kakamega County (Western) and Marsabit County (Eastern) to estimate the proportion of ARI cases seeking care.

Next, virological testing in Kenya was performed at LMH and used to obtain the RSV-associated rates of ARIs. Nasopharyngeal and oropharyngeal (NPOP) samples were systematically tested for the genetic material of respiratory viruses by RT-PCR (polymerase chain reaction) in the base area (Asembo) for all patients meeting the ARI case definition during 2010-2014, thereafter aggregated to annual RSV positivity for scaling to missing years of testing for the base (Nyanza) region. Testing for ARIs was unavailable for other regions (SI Table 1), therefore the ratio of RSV-positivity in ARIs to SARIs in the base region was applied to the SARI testing data of other regions to get the RSV-positivity for ARIs in the other regions.

To estimate RSV-associated SARI in Kenya, first data were collected from children under 5 years of age visiting Kilifi County Referral Hospital (CRH), combined with residency data from participants in the Kilifi HDSS [38]. Similar to ARI, the rate of SARI in the base area was projected to other regions by including risk factors [37] in a linear model and using the estimates for healthcare-seeking behaviour. The proportion of individuals hospitalised with pneumonia from a health utilisation survey was used as a proxy for SARI hospitalisations. RSV-associated SARI was estimated from systematic testing of NPOP samples for RSV in the Coastal and Nyanza Regions. For other regions, some of the data were pooled from five hospitals.

The ratio of out-of-hospital to in-hospital deaths in Kenya was estimated by taking the ratio of these for LMICs provided in Table 4 of Li 2022 [5] (available at [https://www.thelancet.com/action/showFullTableHTML?isHtml=true&tableId=tbl4&pii=S0140-6736%2822%2900478-0](https://www.thelancet.com/action/showFullTableHTML?isHtml=true&tableId=tbl4&pii=S0140-6736(22)00478-0)), in the age brackets shown in Figure 3 of the main text.

**1.2 Incidence data from South Africa: methods and definitions**

The definition for hospitalised SARI was admission to hospital with a physician’s diagnosis of LRTI, with or without fever, and a duration of 10 days or less. For South Africa, there were two principal data sources, similar to the methodology of a previous study [14] on influenza. Surveillance was carried out in two public health outpatient clinics for ILI and in three public hospitals for ALRI in two provinces with a combined catchment area of nearly half a million people in 2015. Respiratory specimens were collected from all enrolled patients (ILI, SARI) and tested for 10 viruses, including RSV. RSV-positive co-infections were included. Secondly, healthcare utilisation surveys (HUS) [40,41] were used for estimates of healthcare-seeking behaviour among individuals with reported ILI or SARI symptoms in the two communities. Similarly to Kenya, risk factors [43,44,52] (exposure to indoor air pollution, crowding, malnutrition, low birth-weight) were taken from the Demographic Health Survey (DHS) to project the SARI rates of the base region to other regions of the country.

For non-severe cases, RSV-associated influenza-like illness (ILI) cases were used. Using the same methodology as for SARI [14], cases were classified as ILI by case report forms for participants enrolled in the surveillance program and samples were transported to and RT-PCR-tested in the National Institute for Communicable Diseases (NICD) for 10 respiratory viruses, including RSV. The South Africa Demographic and Health Survey (DHS) [45] was used to account for risk factors to project local rates across provinces and calculate the national average. Age-specific rates of ILI were calculated from the corresponding SARI rates scaled by the proportion of SARI cases that sought outpatient care before hospitalisation and the ratio of hospital-referred to total ILI consultations [14]. ILI cases were defined as a person presenting with either temperature ≥38C or a history of fever and cough of a duration ≤10 days [53]. This is a narrower definition than the ARI definition in Kenya. To make the two case definitions comparable, we used data on the proportion of ARI cases with fever (23.3%), collected between 2015 and 2017 (2015: 33.3%, 2016: 20.5%, 2017: 16%) in Kenya [49], to broaden the ILI definition (by dividing by the proportion of ARI cases with fever) and align it with ARI. Figures in the main text are by using this broader definition.

Age-specific estimates of in-hospital and out-of-hospital RSV-associated deaths (Figure 3) were derived in a previous publication [15], also described in the accompanying paper by Moyes et al. 2022 [13]. In-hospital mortality was estimated by applying the observed case-fatality ratio to the national number of RSV-associated severe illnesses. Out-of-hospital mortality was estimated using published data on the proportion of deaths that occurred outside of hospital (26% in <5-year-old children).

## ***1.3 Data availability***

The data tables used for each plot in the main text are available in CSV format at

<https://github.com/mbkoltai/RSV-CEA-Kenya-South-Africa/tree/master/output/cea_plots>

in the GitHub repository. Figures can be reproduced with the R script

<https://github.com/mbkoltai/RSV-CEA-Kenya-South-Africa/blob/master/reprod_figs.R>.

Input data files can be found in the folder <https://github.com/mbkoltai/RSV-CEA-Kenya-South-Africa/tree/master/custom_input>. Two estimates for out-of-hospital deaths in Kenya are provided, *deaths_kenya_tidy_adjusted_12_2022.csv* was used in the article.

We recommend running the code by downloading the repository and launching it as a project by the Rproj file [model.Rproj](https://github.com/mbkoltai/RSV-CEA-Kenya-South-Africa/blob/master/model.Rproj).

***2.1 Fitting confidence intervals of efficacy estimates by beta distributions***

We perform a probabilistic sensitivity analysis to account for uncertainties in parameters, including the efficacy of the preventive biologics used. To obtain a probability distribution we can sample from, we use a beta distribution to fit the CI95 values of the efficacy figures reported from clinical trials (SI Table 2, 4). We used the *optim* function in R to find the beta distribution’s two parameters (α,β), sampling through a range of initial values of α and β that satisfy the constraint for the mean efficacy, E(X)=α/(α+β).

In some cases the lower bound of the confidence interval of the efficacy estimate has a negative tail, which cannot be fit by a beta distribution. In these cases we introduced two other fitting parameters, a ‘scaling parameter’ and a ‘shifting parameter’, so that we are fitting an adjusted beta distribution:

*rbeta(α,β)*scaling_parameter + shifting_parameter*

The fits are in SI Table 4. In the case of the efficacy figure ‘RSV LRTI with severe hypoxemia’ for MV, the mean and the confidence interval cannot be well fit simultaneously. Since the fitting is performed using the confidence intervals, the estimate for the mean is lower than the mean in the data (35% and 48%, respectively).

***2.2 Exponential waning model for efficacy figures of MV and mAb***

We fit MV and mAb efficacy figures with an exponential decay model by setting two constraints. First, the half-life (t_0.5_) was fixed to the half-life of antibodies for MV and to mAb serum concentration observed in the clinical trials [7,8]. For MV the half-life of antibodies is 36.5 days (Table S12 in [7]), whereas for mAb serum concentration it is 59.3 days. The second constraint is that the exponential curves need to have a mean value corresponding to the efficacy figures in the 0-90 days (MV) and 0-150 days (mAb) intervals.

The period of protection is denoted as t_dur_ and was set to 90 days in the case of MV and 150 for mAb.

There are two parameters to be determined, one is a scaling constant (c) for the efficacy and the other is the rate of decay (α). The two parameters are then constrained as:

$exp\left( -\alpha t_{0.5} \right)=0.5$ (SI Eq I)

$\frac{1}{t_{dur}}c\int_{0}^{t_{dur}} exp\left( -\alpha t \right)dt$ = VE (SI Eq II)

Reordering, we have for the two parameters:

$\alpha=log\frac{\left( 2 \right)}{t_{0.5}}$ (SI Eq III)

$$c=\alpha\frac{t_{dur}VE}{1-exp\left( -\alpha t_{dur} \right)}$$

In some cases this calculation yields a *c* value above 1 (which would mean a more than 100% efficacy). In this case we fixed *c* to 1, and calculated *α* as

$1=\alpha\frac{t_{dur}VE}{1-exp\left( -\alpha t_{dur} \right)}$ (SI Eq IV)

yielding

${\alpha t}_{dur}+log\left( \alpha\right)=\frac{1}{t_{dur}VE}$ (SI Eq V)

Which we solved numerically for *α.* This calculation leads to half-lives slightly above the values reported in the clinical trials, but the same mean efficacy value over the relevant period.

The values of *c* and *α* for MV and mAb with the different endpoints are shown in SI Table 3.

## ***3. Comparison with parameter estimates in [Li et al 2020]***

## Hospitalisation probabilities in Li et al. [12] were assumed to be identical across age groups (mean value of 8.7%) based on Nokes et al. [55], resulting in an identical age distribution for all hospitalised cases, with uncertainty accounted for by doubling the standard deviation.

Using generalised additive mixed models, the Li et al. analysis inferred an age-specific incidence curve of RSV disease from the data in the global RSV meta-analysis by Shi et al [3]. The incidence curve was calibrated to peak at 6 months of age at a value depending on the country-specific total incidence, which was in the range of 100-150 disease episodes per 1000 person-years. Mean duration of illness and hospital stays were sampled from gamma distributions (means: 11.2 and 5.8 days). Efficacy, duration of protection and cost estimates for MV and mAb are shown in SI Table 2.

We updated these estimates with our hospital-based incidence data, and estimated deaths from CFR estimates from the Kenyan study (accompanying paper by Nyawanda et al [56]) or applying previous estimates (ZAF [14]). . The main difference from Li 2020 is in the age distribution of SARI cases being markedly different in our dataset from that of non-severe (ARI) cases, which changes the age-specific rate of hospitalisations and deaths.
